# Aggression subtypes relate to distinct resting state functional connectivity in children and adolescents with disruptive behavior

**DOI:** 10.1007/s00787-020-01601-9

**Published:** 2020-08-13

**Authors:** Julia E. Werhahn, Susanna Mohl, David Willinger, Lukasz Smigielski, Alexander Roth, Christoph Hofstetter, Philipp Stämpfli, Jilly Naaijen, Leandra M. Mulder, Jeffrey C. Glennon, Pieter J. Hoekstra, Andrea Dietrich, Renee Kleine Deters, Pascal M. Aggensteiner, Nathalie E. Holz, Sarah Baumeister, Tobias Banaschewski, Melanie C. Saam, Ulrike M. E. Schulze, David J. Lythgoe, Arjun Sethi, Michael C. Craig, Mathilde Mastroianni, Ilyas Sagar-Ouriaghli, Paramala J. Santosh, Mireia Rosa, Nuria Bargallo, Josefina Castro-Fornieles, Celso Arango, Maria J. Penzol, Marcel P. Zwiers, Barbara Franke, Jan K. Buitelaar, Susanne Walitza, Daniel Brandeis

**Affiliations:** 1 Department of Child and Adolescent Psychiatry and Psychotherapy, University Hospital of Psychiatry, University of Zurich, Neumünsterallee 9, 8032 Zurich, Switzerland; 2 Neuroscience Center Zurich, University of Zurich and ETH Zurich, Zurich, Switzerland; 3 Department of Psychiatry, Psychotherapy and Psychosomatics and Department of Child and Adolescent Psychiatry, Psychiatric Hospital, MR-Center, University of Zurich, Zurich, Switzerland; 4 Donders Institute for Brain, Cognition and Behaviour, Department of Cognitive Neuroscience, Radboud University Medical Center, Nijmegen, The Netherlands; 5 Donders Institute for Brain, Cognition and Behaviour, Centre for Cognitive Neuroimaging, Radboud University, Nijmegen, The Netherlands; 6 Department of Child and Adolescent Psychiatry, University of Groningen, University Medical Center Groningen, Groningen, The Netherlands; 7 Department of Child and Adolescent Psychiatry and Psychotherapy, Central Institute of Mental Health, Medical Faculty Mannheim/ Heidelberg University, Mannheim, Germany; 8 Department of Child and Adolescent Psychiatry/Psychotherapy, University Hospital, University of Ulm, Ulm, Germany; 9 Department of Neuroimaging, Institute of Psychiatry, Psychology and Neuroscience, King’s College London, London, UK; 10 Department of Forensic and Neurodevelopmental Sciences, Institute of Psychiatry, Psychology and Neuroscience, King’s College London, London, UK; 11 Department of Child Psychiatry, Institute of Psychiatry, Psychology and Neuroscience, King’s College London, London, UK; 12 Child and Adolescent Psychiatry Department, Hospital Clinic of Barcelona, IDIBAPS, Barcelona, Spain; 13 Clinic Image Diagnostic Center (CDIC), Hospital Clinic of Barcelona, Magnetic Resonance Image Core Facility, IDIBAPS, Barcelona, Spain; 14 Child and Adolescent Psychiatry and Psychology Department, Institute Clinic of Neurosciences, Hospital Clinic of Barcelona, CIBERSAM, IDIBAPS, Department of Medicine, University of Barcelona, Barcelona, Spain; 15 Child and Adolescent Psychiatry Department, Hospital General Universitario Gregorio Marañón School of Medicine, IiSGM, CIBERSAM, Universidad Complutense, Madrid, Spain; 16 Department of Human Genetics, Donders Institute for Brain, Cognition and Behaviour, Radboud University Medical Center, Nijmegen, The Netherlands; 17 Department of Psychiatry, Donders Institute for Brain, Cognition and Behaviour, Radboud University Medical Center, Nijmegen, The Netherlands; 18 Karakter Child and Adolescent Psychiatry University Center, Nijmegen, The Netherlands

**Keywords:** Reactive aggression, Proactive aggression, Callous-unemotional traits, Default mode network, Amygdala, Functional connectivity

## Abstract

There is increasing evidence for altered brain resting state functional connectivity in adolescents with disruptive behavior. While a considerable body of behavioral research points to differences between reactive and proactive aggression, it remains unknown whether these two subtypes have dissociable effects on connectivity. Additionally, callous-unemotional traits are important specifiers in subtyping aggressive behavior along the affective dimension. Accordingly, we examined associations between two aggression subtypes along with callous-unemotional traits using a seed-to-voxel approach. Six functionally relevant seeds were selected to probe the salience and the default mode network, based on their presumed role in aggression. The resting state sequence was acquired from 207 children and adolescents of both sexes [mean age (standard deviation) = 13.30 (2.60); range = 8.02–18.35] as part of a Europe-based multi-center study. One hundred eighteen individuals exhibiting disruptive behavior (conduct disorder/oppositional defiant disorder) with varying comorbid attention-deficit/hyperactivity disorder (ADHD) symptoms were studied, together with 89 healthy controls. Proactive aggression was associated with increased left amygdala–precuneus coupling, while reactive aggression related to hyper-connectivities of the posterior cingulate cortex (PCC) to the parahippocampus, the left amygdala to the precuneus and to hypo-connectivity between the right anterior insula and the nucleus caudate. Callous-unemotional traits were linked to distinct hyper-connectivities to frontal, parietal, and cingulate areas. Additionally, compared to controls, cases demonstrated reduced connectivity of the PCC and left anterior insula to left frontal areas, the latter only when controlling for ADHD scores. Taken together, this study revealed aggression-subtype-specific patterns involving areas associated with emotion, empathy, morality, and cognitive control.

**Electronic supplementary material:**

The online version of this article (10.1007/s00787-020-01601-9) contains supplementary material, which is available to authorized users.

## Introduction

Aggression is defined as behavior aimed to harm other persons or objects. In young individuals, aggression dimensions partially undercut the normative diagnostic categories [1]. Two clinical manifestations within the disruptive behavior disorder (understood as broad hostile and defiant behaviors), oppositional defiant disorder, and conduct disorder are among the most common psychiatric disorders in childhood and adolescence [2]. They are defined by angry and vindictive behaviors and violating rules, norms, and rights, respectively [3]. Additionally, callous-unemotional traits are reported in a significant percentage of children and adolescents with disruptive behavior disorder [4] and are characterized by callousness, uncaring, and unemotional dimensions [5,6]. Within this complexity of phenotypic manifestation in child and adolescent psychopathology, reactive and proactive aggression (RA and PA, respectively) pertain to distinct functions of aggression, reflecting impulsive and instrumental behaviors, accordingly [7]. On the neural level, differentiation between these two subtypes in humans remains largely unexplored. Yet, there is substantial evidence from animal models and research on neurotransmitters that both forms tap into distinct neural circuits and are linked to acute responses to a threat (RA) or a self-initiated, predatory action (PA) [8].

Among human neuroimaging paradigms, the investigation of brain activity during a resting state [9] has the potential to penetrate clinical practice [10]. It seems especially well-suited to enhance our understanding of the neural foundation of disruptive behavior in children and adolescents. The procedure can be performed in individuals unable to cooperate during cognitive tasks. It targets spontaneous brain activity and delivers biological metrics, such as functional connectivity, which was found to identify inter-individual differences [11, 12]. For example, recent functional magnetic resonance imaging (fMRI) studies of resting state in conduct disorder have reported both increases and decreases in functional connectivity or activity depending on the network. Specifically, the brain regions involved included the amygdala and insula, parts of the salience network (SN) [13–15], as well as the default mode network (DMN) [16, 17]. So far, only a few resting state studies have evaluated callous-unemotional traits. Decoupling between the DMN and fronto-parietal network was found to increase with expression of callous-unemotional traits, indicative of dissociation between cognitive control and a social understanding of others [18].

On the other hand, within-network DMN connectivity was shown to increase with higher callous-unemotional traits, suggesting alterations in self-referential processing [19]. Moreover, the interpersonal/affective dimensions were found to depend on DMN connectivity [20]. Additionally, in male youths with conduct disorder, interpersonal traits were correlated with distinct amygdala subregional connectivity with regions located in the SN and DMN [14]. Compared to male youths with conduct disorder who scored low on callous-unemotional traits or healthy controls, juveniles with conduct disorder who scored high on callous-unemotional traits showed increased amygdala connectivity with a frontal section of the DMN [15]. The findings mentioned above suggest that brain connectivity involving these two networks may reflect the clinical manifestation.

Comorbid attention-deficit/hyperactivity disorder (ADHD) symptoms are frequently present in conduct problems [21], associated with poorer responses to treatments [22], and linked to overlapping neural deficits in prefrontal and limbic areas [23]. The severity of ADHD symptoms was also linked to enhanced functional density [24] and other alterations [25, 18, 26] involving the DMN. Previously, the connectivity between core DMN regions, i.e., the anterior medial prefrontal cortex (amPFC) and the posterior cingulate cortex (PCC), was found to be reduced in male adolescents with conduct disorder compared to healthy controls after controlling for ADHD symptoms, as ADHD symptoms were positively correlated with the connectivity of the DMN [17]. Nevertheless, these fMRI studies largely neglected distinct manifestations of aggression [7]. Despite considerable behavioral research accentuating the differentiation between RA and PA—with evidence for both being correlated [27], but also associated with different behavioral symptoms [28]—no investigation of resting state functional connectivity to date has addressed their underlying mechanisms in children and adolescents with disruptive behavior. Additionally, most of the studies have not included healthy controls and discarded additional aggression dimensions.

In consideration of the above rationale, this study examines the distinct patterns of resting state functional connectivity associated with RA and PA, along with callous-unemotional traits, in children and adolescents with disruptive behavior. We applied a well-established seed-to-voxel approach, which computes cross-correlations within time-series data derived from blood-oxygen level dependent (BOLD) signals in a specific seed with the rest of the brain. This approach enables the detection of functional connections with any voxel or cluster of voxels lying within or outside of any specific network. Based on the findings of the altered connectivity of brain areas in the DMN [16, 17, 29, 24] and SN [14, 15], we defined the functionally relevant regions of interest to probe these two networks, i.e., within the PCC, amPFC, bilateral anterior insula, and bilateral amygdala. Our hypothesis assumed distinct connectivity patterns for proactive/reactive aggression and callous-unemotional traits [14, 15, 19]. To replicate earlier findings on case–control differences, we expected to observe reduced connectivity in aggressive cases compared to healthy controls and increased connectivity with higher ADHD scores [17, 24]. Overall, we hypothesized that cases would differ in connectivity and correlations with clinical symptoms, with the involvement of areas linked to the processing of emotion, empathy abilities, and cognitive control, such as the PCC—the prefrontal cortex, amygdala–precuneus, the anterior insula–frontal areas, and/or limbic areas.

## Methods and materials

### Participants

The participants in the current study were part of the joint EU-MATRICS (https://matrics-project.eu) and EU-Aggressotype (https://www.aggressotype.eu) projects. Both multi-center research initiatives aim to gain new insights into the mechanisms underlying aggression, especially by identifying biological and behavioral correlates for subtypes of aggression. The applied methods include animal models, genomics, epigenomics, transcriptomics, neurochemistry, and human neuroimaging. Children and adolescents aged 8–18 years were recruited from resident hospitals, ambulatories, and eligible (boarding) schools. A total of 207 individuals (*n* = 150 males) comprising cases (*n* = 118) and healthy controls (*n* = 89) were included from nine different sites in Europe (mean age ± standard deviation (SD): 13.30 ± 2.60 years). As the main goal was to conduct aggression subtype-specific analyses, recruitment focused on including cases presenting with a diagnosis of conduct disorder and/or oppositional defiant disorder and/or aggression scores in a clinical range (*T* > 70) according to the Child Behavior Checklist (CBCL), Youth Self Report (YSR), or Teacher Report Form (TRF) [30]. Cases were required additionally to take no medication or be on stable medication for at least 2 months. Further exclusion criteria were as follows: a primary DSM-5 diagnosis of depression, anxiety, psychosis, or bipolar disorder for cases, and a DSM-5 diagnosis or clinically relevant scores in the CBCL, YSR, or TRF for healthy controls. Additional exclusion criteria for all participants included the following: standard contraindications for MRI scanning (i.e., braces, metal medical implants), an anxiety score > 8 on a Visual Analogue Scale ranging from 1 to 10 (because anxiety caused by the scanner could impair data quality and alter neural activity [31]), and an IQ score lower than 80. All individuals had sufficient native language skills based on the country of the assessment. Participants and their parents or legal representatives gave written informed consent. Each site obtained ethical approval separately.

### Clinical assessments

The semi-structured interview Kiddie Schedule for Affective Disorders and Schizophrenia Present and Lifetime version (K-SADS-PL) [32] was used to assess the diagnostic criteria for all participants, as conducted by trained psychologists or trained and supervised interns based on the reports of participants and their parents, who were interviewed separately. The self-reported Reactive Proactive Aggression Questionnaire [33] measured the RA and PA forms of aggression. To assess callous-unemotional traits, the parents filled out the Inventory of Callous-Unemotional traits (ICU) [5, 34], which consists of three subscales assessing callousness, uncaring, and unemotional behaviors. ADHD symptoms were evaluated using the inattention, hyperactivity, and impulsivity counts of the K-SADS-PL instrument. Further details on the clinical assessment are provided in the Supplemental Information.

### Image acquisition

For data acquisition, six sites used Siemens 3 T (T) scanners, two sites used Philips 3 T scanners, and one site used a GE 3 T scanner (see Supplemental Table S1 and Table S2 for detailed scanner specifications). T1-weighted anatomical scans and T2*-weighted echo-planar resting state functional imaging were performed with predominantly similar parameters across sites (TR 2.45 s or less, at least 32 slices). The average acquisition time was 8 min 25 s. The participants were instructed in a standardized fashion to lie still, look at a white crosshair presented against a black background, not think about anything specific, and let their mind wander (to avoid constraining spontaneous thoughts [35]).

### Data preprocessing

Preprocessing was conducted using SPM12 (Welcome Trust Centre for Neuroimaging, UCL, United Kingdom; https://www.fil.ion.ucl.ac.uk/spm) and the SPM-based CONN toolbox v17.b (https://www.nitrc.org/projects/conn). The initial steps included realignment, unwarping, and slice timing correction. Subsequently, the multi-echo data were linearly weighted by their echo time (TE) using MATLAB (The MathWorks, MA, USA): *Y*_4_ = (*Y*_1_ × TE_1_/TE_sum_) + (*Y*_2_ × TE_2_/TE_sum_) + (*Y*_3_ × TE_3_/TE_sum_), where *Y* is the echo file and TE is the echo time. Data were further normalized based on the Montreal Neurological Institute (MNI) brain template [36], followed by an artifact-detection step using the Artifact Detection Tools (ART-toolbox, https://www.nitrc.org/projects/artifact_detect), smoothing with a 6-mm full-width at half-maximum Gaussian kernel, and segmentation to derive the white matter and cerebrospinal fluid parameters. The aCompCor strategy [37] implemented in CONN was applied during denoising to reduce the effect of physiological and motion-related noise [38]. Specifically, after identifying principal components of the subject-specific white matter and cerebrospinal confounds, aCompCor extracted the estimated time-series by adding them as regressors. Additionally, the movement parameters derived from realignment were added as regressors. After denoising, the initial hemodynamic response function signal aberrations were removed. Compared to the global signal regression method, aCompCor shows higher sensitivity and specificity regarding positive correlations and anticorrelations [39]. To circumvent the influence of low-frequency drifts and high-frequency noise, including heart rate and respiration [38], we applied temporal band-pass filtering (0.008–0.9 Hz).

### Motion censoring

Given that head motion can lead to changes in BOLD signals [40] and that our cases presenting with externalizing disorders included comorbid ADHD symptoms, we used the threshold for excessive motion applied in recent fMRI studies of adolescents with ADHD [41]. This approach led to the exclusion of ten cases with a root mean square framewise displacement (RMS-FD) of > 0.95 mm. Moreover, a very conservative threshold for detecting functional outlier scans was applied (> 3 mm standard deviations from the observed global BOLD signal and > 0.5 mm composite scan-to-scan motion). Twelve participants were excluded because of the missing or insufficient quality of structural scans, and 14 more were excluded based on image artifacts. Additional sensitivity analyses implementing even stricter motion censoring are provided in the Supplemental Information.

### Regions of interest

Six seeds were chosen, based on their functional relevance for aggression, to probe the DMN and SN [42]. The PCC and amPFC seeds (each including 257 voxels) were created in the MarsBar toolbox (v0.44) (https://marsbar.sourceforge.net) and centered on coordinates recently used [17] and provided by Andrews-Hanna et al. in their (functional) study of the DMN [43]. The bilateral amygdala and bilateral anterior insula (including 92 and 118 voxels per seed, respectively) are the activation regions of interest derived from the Face Matching Task (negative faces versus shapes) applied and published previously [44]. Given the typically differing connectivities between various parts of the insula and its involvement in emotional processing [45], only the anterior section was selected. Task-related seeds were also chosen to allow future comparisons between the resting state and task within the same study.

### Functional connectivity analysis

Seed-based connectivity analyses were performed using the CONN toolbox. First-level analysis computed Pearson’s correlation coefficients between the time course of previously denoised BOLD-signals from seed and whole-brain voxels. After Fisher’s transformation to normally distributed *z*-scores, general linear model (GLM) analyses were conducted. Besides site, added as a dummy-coded covariate of no interest in second-level analyses, age, sex, IQ, medication, and handedness were also controlled for based on previous reports of their possible influences on the connectivity of the DMN [46]. A random effects analysis of covariance for group comparisons included further analyses, adding ADHD symptoms as additional covariates of no interest, based on previous reports on the importance of considering ADHD symptoms to differentiate connectivity patterns between cases and controls [17, 26]. Supplemental sensitivity analyses were performed, excluding cases that did not meet the DSM-based diagnosis. Linear regressions separately tested the association between RA, PA, and ICU scores with connectivity values in cases. Supplemental exploratory dimensional analyses (limited by the small and unmatched subsamples) were also performed, in consideration of the putative role of age and sex. The results of the seed-based analyses are reported at thresholds of *p* < 0.001 and cluster-corrected *p*-FWE < 0.008 (= 0.05/6, using additional Bonferroni corrections for the number of seeds).

## Results

### Sample characteristics

Out of 118 cases, 48 had a diagnosis of oppositional defiant disorder, 25 of conduct disorder plus oppositional defiant disorder, and 7 of conduct disorder. Seventy-seven cases presented with a clinically relevant score (*T* > 70) on the aggression or rule-breaking behavior subscales of the CBCL and 41 cases on both subscales. Thirty-eight cases had an aggression score in the clinical range but no DSM diagnosis (Table 1). While cases and controls were matched for age and handedness, there were more males than females in the case group (99–19); cases also exhibited a lower IQ than healthy controls and showed a wide distribution of RA and PA levels, callous-unemotional traits, and ADHD symptoms. The proportion of controls relative to cases differed across participating sites. For the distribution of diagnoses, aggression scores, medication, and demographic variables by site, please see Supplemental Table S3.

**Table 1:** Sample characteristics

Characteristic	Cases (*n* = 118)	HC (*n* = 89)	Test statistic	*p*
Age (years)	13.23 ± 2.68	13.40 ± 2.49	*t(*205) = − 0.45	0.65
Sex, m/f	99/19	51/38	*χ*^2^ = 17.98	< 0.001
IQ^a^	100.78 ± 11.00	106.64 ± 10.42	*t*(195) = − 3.81	< 0.001
Handedness, left/right	16/95	10/77	*χ*^2^ = 0.37	0.55
CD plus ODD diagnosis^b^	25			
ODD diagnosis^b^	48			
CD diagnosis^b^	7			
ADHD diagnosis^b^	29			
CBCL—Aggression T-score	74.46 ± 10.10	51.77 ± 6.19	*t*(197) = − 12.78	< 0.001
CBCL—Rule-breaking T-score	69.00 ± 12.14	51.69 ± 6.72	*t*(196) = − 20.54	< 0.001
K-SADS—inattention	3.33 ± 2.91			
K-SADS—hyperactivity	1.66 ± 1.91			
K-SADS—impulsivity	1.08 ± 1.20			
ICU—total score	33.68 ± 10.16	21.00 ± 8.70	*t*(196) = − 9.44	< 0.001
ICU—callousness	12.00 ± 6.11	4.00 ± 3.44	*U* = 2278.00	< 0.001
ICU—uncaring	17.00 ± 3.93	10.41 ± 5.07	*U* = 2445.00	< 0.001
ICU—unemotional	7.17 ± 3.31	5.22 ± 2.75	*t*(189) = − 4.37	< 0.001
RPQ—reactive aggression	12.55 ± 5.09	5.00 ± 3.48	*U* = 1296.50	< 0.001
RPQ—proactive aggression	3.00 ± 5.01	0.82 ± 1.45	*U* = 1807.50	< 0.001
Medication use^c^	70			
Stimulants	52			
Neuroleptics	18			
Antidepressants	2			
Mean RMS-FD	0.12 ± 0.17	0.09 ± 0.18	*U* = 4134.50	< 0.01

Values are means or, for non-normal distributions, medians ± SD or counts

*ADHD* attention-deficit hyperactivity disorder, *CBCL* Child Behavior Checklist, *CD* conduct disorder, *HC* healthy controls, *ICU* inventory of callous-unemotional traits, parent report, *K-SADS* Kiddie Schedule for Affective Disorders and Schizophrenia, *ODD* oppositional defiant disorder, *RMS-FD* root mean square framewise displacement, *RPQ* Reactive and Proactive aggression Questionnaire

^a^IQ estimated based on four sub-tests derived from the Wechsler Intelligence Scale for Children IV

^b^Diagnoses derived from the Kiddie Schedule for Affective Disorders and Schizophrenia Present and Lifetime version

^c^Medication use according to parental or clinical reports

### Group differences in functional connectivity

**Fig. 1:**
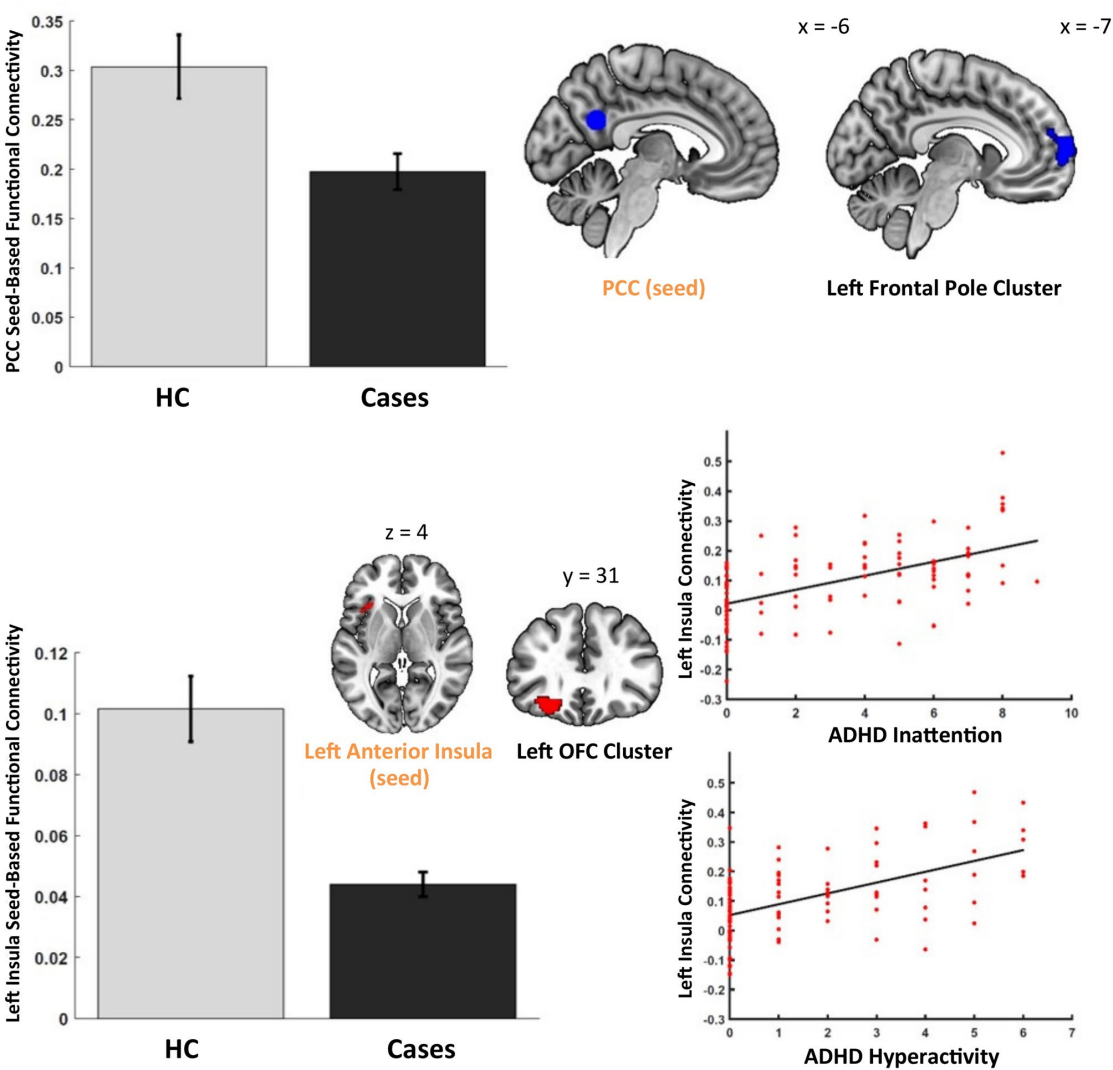
Significant results from the case–control functional connectivity analysis. The bar charts show a reduced seed-to-voxel connectivity pattern for cases compared to healthy controls (HC). The seeds and corresponding projections are mapped onto a brain diagram. The scatterplots depict the main effect of ADHD inattention, and hyperactivity counts in cases plotted against the connectivity values between the seed in the left anterior insula and projection in the left orbito-frontal cluster

Compared with controls, cases demonstrated reduced connectivity of the PCC seed with a projection cluster including the left frontal pole [*t*(197) = 5.46, cluster-size *p*-FWE < 0.008, peak uncorrected *p* < 0.001, *β* = 0.10] (Fig. 1, Supplemental Table S4). For the seed in the left anterior insula, after taking into account the ADHD symptoms, cases showed diminished connectivity with a cluster extending from the orbitofrontal cortex (OFC) to the frontal pole [*t*(194) = 5.07, cluster-size *p*-FWE < 0.008, peak uncorrected *p* < 0.001, *β* = 0.10) (Fig. 1, Supplemental Table S4). These analyses were controlled for site. Furthermore, a subsequent analysis revealed a positive correlation between ADHD inattention and hyperactivity counts and the strength of connectivity within cases for this left anterior insula–frontal cluster effect, however, at a lower significance threshold [*t*(85) > 5.27, all cluster-size *p*-FWE < 0.05, peak uncorrected *p* < 0.001, *β* = 0.04–0.09] (Fig. 1). There were no other statistically significant findings in these analyses.

### Functional connectivity correlates of reactive versus proactive aggression

**Fig. 2:**
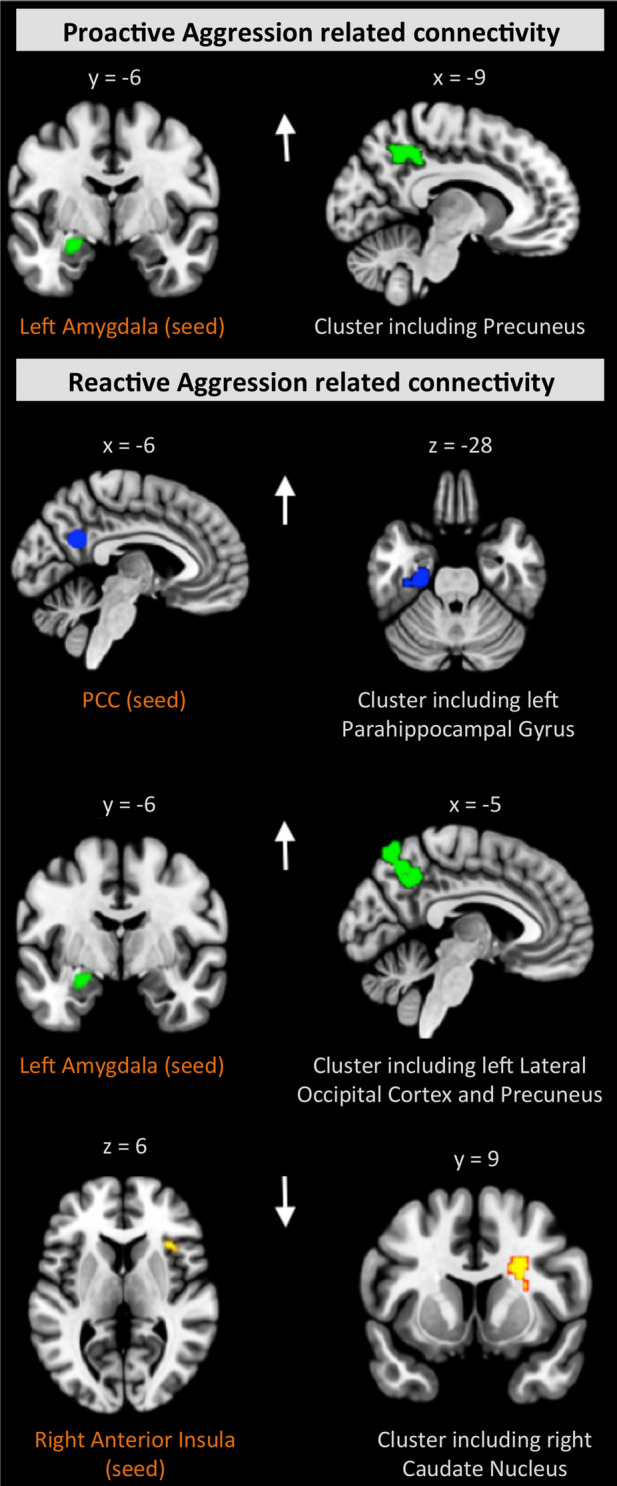
Distinct RA- and PA-related connectivity patterns within cases. There was only one partly overlapping connectivity pattern related to both RA and PA for the seed in the left amygdala. The seed regions are depicted in the left panel, with corresponding projections shown in the right panel. The results are FWE-corrected, *p* < 0.008. The arrows reflect the effect direction (hyper- and hypo-connectivity)

Our analysis testing for correlates of RA and PA using the previously applied six seeds yielded subtype-specific results (Fig. 2, Supplemental Table S6). Specifically, PA scores were positively associated with the connectivity strength between the seed in the left amygdala and the precuneal cluster. Moreover, cases with higher RA scores also showed increased connectivity of the seed in the PCC with a cluster extending from the left parahippocampal gyrus to the left inferior temporal gyrus. Furthermore, RA scores were positively correlated with connectivity between the left amygdala seed and a projection cluster extending from the left lateral occipital cortex to the precuneus. There was also a negative association between RA scores and connectivity between the right anterior insula seed and a cluster localized in the right caudate nucleus (all cluster-size *p*-FWE < 0.008, *β* = 0.04–0.05). The above findings remained significant at an uncorrected *p* < 0.01 threshold after applying the more stringent excessive motion criterion (see sensitivity analyses, Supplemental Table S11).

### Functional connectivity correlates of callous-unemotional traits

**Fig. 3:**
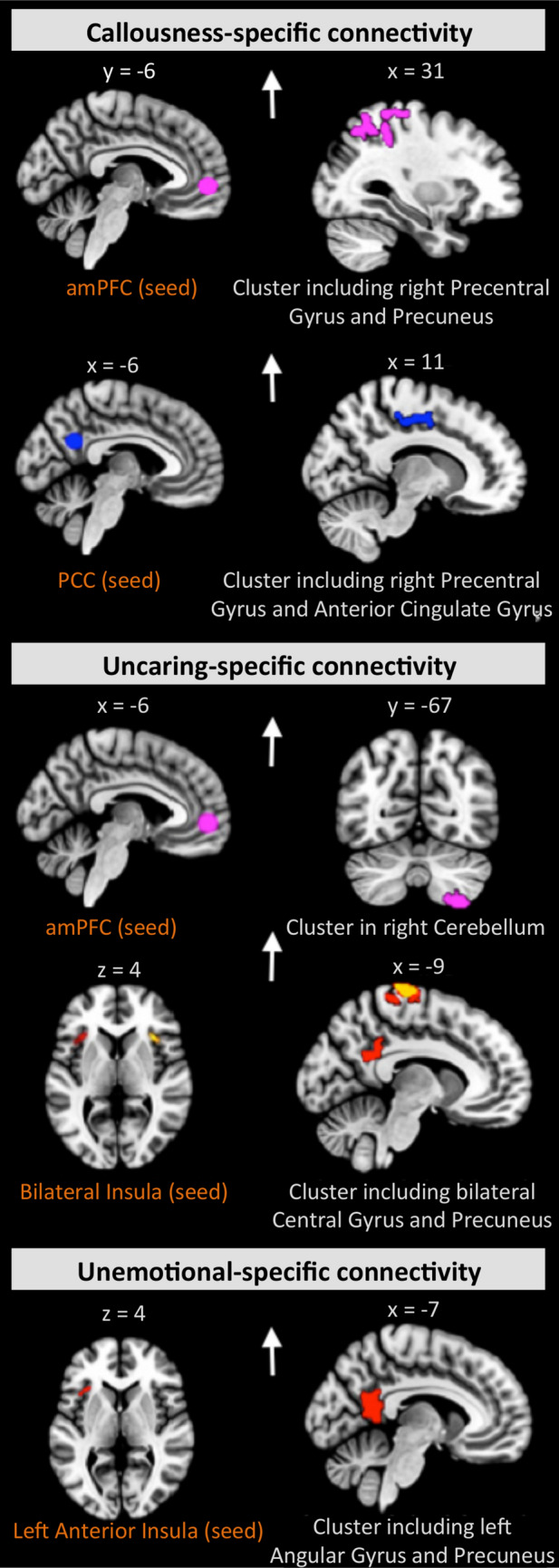
Differing connectivity patterns related to scores on the callousness, uncaring, and unemotional dimensions within cases. The seed regions are depicted in the left panel, with corresponding projections shown in the right panel. The results are FWE-corrected, *p* < 0.008. The arrows reflect the effect direction (i.e., hyper- and hypo-connectivity)

Our analysis testing for correlates of callous-unemotional traits revealed positive associations with distinct seed-based connectivity patterns within cases (Fig. 3, Supplemental Table S7). Specifically, the callousness scores were linked to connectivity between the PCC and a cluster in the right precentral gyrus and cingulate areas, as well as between the amPFC and a region including the right precentral gyrus and precuneus. Uncaring behavior scores were associated with connectivity between the following pairs of regions: the amPFC and right hemispheric cerebellar regions, the left anterior insula and precuneal and cingulate clusters, and the right anterior insula and left central gyrus. Finally, unemotional-specific connectivity was identified for the seed in the left anterior insula and a projection in the precuneus together with the angular gyrus (all cluster-size *p*-FWE < 0.008, *β* = − 0.06 to 0.08) (Fig. 3, Supplemental Table S7).

### Sensitivity analysis for cases without a DSM diagnosis

Complementary sensitivity analyses for case–control comparisons were conducted excluding 38 cases without a DSM diagnosis. The results align with the analysis without the exclusion. Cases exhibited a reduced connectivity of the PCC to the left frontal pole (*M* = 0.20) compared to controls (*M* = 0.30; *U* = 3250,* p* < 0.001). The effect was present after the exclusion of cases without a diagnosis (*U* = 2297, *p* < 0.001), with similar results for cases (*M* = 0.20) and controls (*M* = 0.30). After an additional correction for ADHD scores, cases showed a diminished left anterior insula connectivity (*M* = 0.05) compared to controls (*M* = 0.10; *U* = 2690,* p* < 0.01). Details on all the performed sensitivity analyses are provided in the Supplemental Information.

### Effects of covariates on group differences

For the seed in the PCC, the results withstood controlling for site, age, sex, and IQ at a less conservative significance threshold (cluster-size *p*-FWE < 0.05) and application of a more stringent excessive motion criterion (see Supplemental Table S11 for details on the sensitivity analyses). However, when tested using a full list of covariates, i.e., site, age, sex, IQ, medication, and handedness, no significant results were observed. For the seed in the left anterior insula, the group difference withstood correction for site, age, sex, IQ, and handedness. Additionally, to explore the influence of the covariates as main predictors (sex, IQ, medication, and handedness) on group differences, we conducted sensitivity analyses. These analyses showed that none of these covariates had a significant impact on the relationship between aggression and functional connectivity (all *p* > 0.05), except as described for the exploratory analyses of age and sex.

### Dimensional analysis of the whole sample

The analysis of functional connectivity correlates in the whole sample showed a decreased connectivity between the PCC and angular gyrus for PA and overlapping increased connectivity of the left amygdala with the precuneus for both PA and RA (Supplementary Table S13–S14, Figure S1), paralleling the effect in cases.

### Age as a moderator

The dimensional analysis was also rerun with age as a moderator between the connectivity values and clinical scales. Three significant interactions were found only in cases, pointing to increased associations with higher age for the left anterior insula–precuneus and left anterior insula–pre-/post-central gyri (both for unemotional traits) as well as the right anterior insula–post-central gyri and the total score from the inventory of callous-unemotional traits (see Supplement pages 25–26 and Figure S2 for details).

### Exploratory sex-related analyses

Further, sex by group interactions (after controlling for all other covariates) was found for the connectivity patterns between the left insula and the right superior temporal gyrus. Specifically, female cases showed higher connectivity than female controls, when compared to male participants, who showed lower connectivity in cases than in controls. In the whole sample, there was also lower connectivity in males than females between the amPFC and the superior lateral occipital cortex and between the left insula and the supplementary motor areas and a cluster in the temporal pole. Other effects largely coincided with non-labeled regions (Supplemental Tables S18–S20). There were some tentative sex-dependent effects in relation to the aggression scales and connectivity in cases, being largest for the seed in the right insula and PA and RA (Supplemental Table S21). Sex did not moderate the interaction among the ADHD symptoms (inattention, hyperactivity), group, and connectivity in our main finding (i.e., for the left anterior insula and the left OFC; all *p* > 0.05).

## Discussion

The present multi-center study investigated aggression subtype-specific functional brain connectivity in a sizeable sample of children and adolescents with disruptive behavior, including those with diagnoses of conduct disorder and oppositional defiant disorder. In line with our hypothesis, RA and PA were related to distinct couplings of brain regions implicated in emotion, empathy, and cognitive control. Specifically, PA scores were positively associated with connectivity between the amygdala and precuneus, while RA scores were associated with the increased coupling of the PCC, amygdala, and anterior insula with the (para-)limbic and precuneal clusters. In terms of overlaps, both aggression subtypes involved increased connectivity of the left amygdala with the precuneus. Impaired amygdala function and its connections seem to exert crucial effects on both the neural threat circuitry related to a higher risk for RA and in moral behavior, increasing the risk for PA [47]. The connectivity of the precuneus was closely related to impulsivity in a recent study [24], which represents an important observation given the association of impulsivity and RA [28]. Interestingly, the precuneus cluster in our study was much larger for RA than for PA. Additionally, the amygdala-related projection areas extended to the occipital regions for RA, which may further underpin subtype-specific differentiation, in line with earlier behavioral results [28]. The RA-specific increased connectivity from the PCC to paralimbic and limbic regions aligns with the finding of their abnormal functioning in psychopathy [48]. Specifically, for RA, we found positive functional wiring of the anterior insula with the right caudate nucleus, a region involved with integration of performance and cognitive control [49]. It is known that the neural circuits including the anterior insula regulate the responses to frustration and perceived social provocations [50].

In addition to the PA-/RA-related results, different connectivity patterns were also found for callous-unemotional traits, corroborating previous neural [19, 52, 14, 15, 48] and behavioral findings [52]. As a general remark, our cases exhibited higher connectivity with higher callous-unemotional traits, in the absence of significant negative associations, which is in line with the majority of earlier resting state research literature investigating adolescents [19, 14, 15] and adults [51, 53]. These traits were related in our study to different DMN- and SN-based hyper-connectivities with projection clusters in frontal, parietal, cingulate, precuneal, and cerebellar areas. Specifically, both uncaring and unemotional traits were linked to a stronger coupling between the anterior insula with the precuneal and cingulate clusters, extending to the central and angular gyrus. A recent meta-analytic review confirmed that the right anterior insula and its connectivity are implicated in the evaluation of feelings and the left anterior insula in the expression of anger [54]. Altered insular and cingulate functioning has been previously linked to moral reasoning in adult psychopathy [55]. The results from our study pointed to an association between callousness and uncaring traits and the connectivity patterns involving the precentral gyrus, which has been earlier identified as a hot spot region in adolescents with callous-unemotional traits [19, 18] and psychopathic adults [50, 53, 56, 48]. The DMN connectivity effects reported here may also suggest uncaring and callousness dimension-specific alterations in (affective) self-referential processes [43]. Notably, altered connectivity in the DMN has been repeatedly shown in children and adolescents with callous-unemotional traits [19, 18] and previously in adult psychopathy [53, 57, 58]. The precuneus, as part of the DMN, contributed to classifying adults with antisocial personality disorder in a previous study [58]. In addition, the overlapping connectivity in the insula projecting to the precuneal and cingulate areas for uncaring and unemotional behavior scores may reflect the variance overlap between both constructs [34]. Intriguingly, we did not find any overlapping connectivity patterns between callous-unemotional traits and RA or PA, despite well-known behavioral commonalities [34, 52] and the behavioral correlation of PA scores with callous-unemotional traits in our sample. Also, contrary to previous reports, no effect on amygdala connectivity was found for these traits [14, 15, 59]. This result aligns with previous findings suggesting neural alterations beyond (para-)limbic regions in adult psychopathy [55]. Interestingly, our most recent study indicated differences in amygdala activity and skin conductance during an emotion-processing task between cases and typically developing controls, with a moderating effect of callous-unemotional traits [60]. Overall, the specific connectivity patterns between seeds and projections identified in the present investigation of callous-unemotional traits, point to mechanisms linked to emotion, empathy, moral, and self-referential processes, which are very often impaired in youth with disruptive behaviors [61].

Compared to healthy controls, cases exhibited diminished connectivity of the PCC (a key brain hub in the DMN) with a cluster in the left frontal pole. By additionally controlling for ADHD symptoms in the analysis, we further identified decreased connectivity between the left anterior insula (a key brain hub of the SN) and a left hemispheric frontal cluster. Abnormal connectivity of the PCC has been demonstrated in male adolescents with conduct disorder [17–19] and may suggest impaired self-referential processes [43]. Our results also support the crucial role of comorbid ADHD symptoms previously shown for the DMN in male adolescents with conduct disorder [17] and extend these findings to the seed-based approach centered in the major DMN and SN areas with whole-brain projections. In line with previous studies [17, 19, 24], cases exhibited an ADHD score-related increase in connectivity, which further corroborates the recent finding of overlapping deficit functioning in ADHD and disruptive behavior disorders [23]. However, the observed group differences only withstood post hoc analysis when other covariates were not controlled for or when a lower statistical threshold was applied. Therefore, a somewhat cautious interpretation of these specific results is merited.

The present study has some limitations. First, it is worth noting that our 38 aggressive cases without a DSM diagnosis of conduct disorder and/or oppositional defiant disorder exhibited lower PA scores compared to cases with a DSM diagnosis. However, subsequent sensitivity analyses led to comparable results of case–control group comparisons when excluding these cases without a diagnosis. Second, we did not consider the self-reports of callous-unemotional traits. Yet, a recent study suggests a higher criterion validity for parent-reported callous-unemotional traits compared to self-reports and teacher reports [62]. Third, the distribution of cases and controls across sites was unbalanced, and both groups were not matched for sex, IQ, or the number of participants. Nevertheless, the sensitivity analyses conducted showed no significant influence of IQ, medication, or handedness on the connectivity of cases compared to controls. Fourth, data for the current study were collected at different sites with equipment from different scanner manufacturers and with partly varying scan parameters, which affected data homogeneity and limited our study power. On the other hand, the larger sample size and the multi-center approach might have increased the reliability and generalizability of our results. Finally, sex-dependent effects were supported by our exploratory analyses, in line with earlier brain imaging studies on aggressive behavior [63]. They should be investigated more in-depth in the future in study samples with comparable sex distributions.

By evaluating the effect of RA and PA along with callous-unemotional dimensions, we have extended current knowledge on disruptive behavior disorder and largely understudied distinct manifestations of aggression. These results provide a rationale for treating aggression not as an entity, but as an array of distinct subtypes that can be differentiated on clinical and neural grounds. In particular, therapies for children and adolescents with disruptive behavior may be improved through careful identification of such distinct aggression subtypes, a better understanding of their neural correlates, and the development of interventions counteracting this aberrance. An example of practical application may be real-time fMRI neurofeedback targeting selected brain regions in specific subtypes of aggression and to learn self-regulating brain activity. Moreover, our results may further point to dissociable developmental trajectories, as some of the observed brain areas are also related to adult psychopathy. Notably, RA and PA differentially predict later conduct problems [64], and future studies applying a longitudinal design will be best positioned to track further such effects across time.

## Electronic supplementary material

Below is the link to the electronic supplementary material.

Supplementary file1 (DOCX 2346 kb)
