# Supplementary material for: Aggression subtypes relate to distinct resting state functional connectivity in children and adolescents with disruptive behavior
Source: Eur Child Adolesc Psychiatry. 2020 Aug 13;30(8):1237–49. doi: 10.1007/s00787-020-01601-9 (PMC8310860; doi:10.1007/s00787-020-01601-9)
Supplement: Supplementary file 1 — Supplementary file1 (DOCX 2346 kb) [file 787_2020_1601_MOESM1_ESM.docx]

**Aggression Subtypes Relate to Abnormal Resting State Functional Connectivity in Children and Adolescents with Disruptive Behavior**

**Supplemental Information**

**Supplementary Methods and Materials**

**Participants**

The current analyses included 207 children and adolescents. One hundred eighteen cases exhibited disruptive behavior (mean age = 13.23, *SD* = 2.68), and 86 were age- and handedness-matched healthy controls (mean age = 13.40, *SD* = 2.49). Among the cases, 25 had a DSM-5 diagnosis of conduct disorder and oppositional defiant disorder, 48 of oppositional defiant disorder, 7 of conduct disorder, and 77 cases (additionally) presented with an aggression score in the clinical range (T > 70) on the aggression or rule-breaking behavior subscales according to the Child Behavior Checklist (CBCL), with 41 cases meeting this threshold on both subscales. Thirty-eight cases had an aggression score in the clinical range but no DSM-diagnosis. Out of 29 cases with comorbid ADHD diagnosis, 15 cases had a diagnosis of oppositional defiant disorder only, with a single case of conduct disorder only and 8 having a diagnosis of oppositional defiant disorder plus conduct disorder. None of our controls met a diagnosis of ADHD nor ADHD inattention or hyperactivity/impulsivity syndromes according to the Kiddie-Schedule for Affective Disorders and Schizophrenia (K-SADS).

**Study sites**

Participants were recruited across nine different sites in Europe:

- Radboud University Medical Center and the Donders Center for Cognitive Neuroimaging Nijmegen The Netherlands;
- Department of Neuroscience University Medical Centre Groningen The Netherlands;
- Central Institute of Mental Health Medical Faculty Mannheim/Heidelberg University Mannheim Germany;
- University of Ulm Department of Child and Adolescent Psychiatry/Psychotherapy and Department of Psychiatry III Ulm Germany;
- Department of Child Psychiatry and the Centre for Neuroimaging Sciences Institute of Psychiatry Psychology and Neuroscience King`s College London London England;
- Institut d'Investigacions Biomèdiques August Pi i Sunyer and Hospital Clinic de Barcelona Barcelona Spain;
- Instituto de Investigación Sanitaria Gregorio Marañón Child and Adolescent Psychiatry Department and Ruber International Hospital Madrid Madrid Spain;
- Department of Child and Adolescent Psychiatry and Psychotherapy University Zurich and MR center Psychiatric University Hospital Zurich Switzerland;
- IRCCS Santa Lucia Foundation Rome, Italy.

Twelve participants (11 cases, one control) were excluded owing to missing (*n* = 2) or insufficient quality of the T1-weighted anatomical scans (*n* = 10) from our original data set of resting state sequences from 141 cases and 92 healthy controls. Moreover, 12 cases and two controls were excluded owing to image artifacts and 10 cases owing to excessive motion. Finally, 118 cases and 89 controls, both males (*n* = 150) and females (*n* = 57) aged 8 to 18 years, were included in the resting state functional connectivity analysis.

**Assessment Tools and Study Procedure**

Diagnostic assessments and MR measurements took place on different dates to minimize the burden on participants. During the first appointment, the parents/primary caregivers and children/youths were assessed separately by trained (clinical) psychologists or interns with the semi-structured interview Kiddie-Schedule for Affective Disorders and Schizophrenia Present and Lifetime version (K-SADS-PL) [1]. The full supplementary module of the specific disorder followed positively answered questions. The diagnoses resulted from self- and parent-reports. If not already completed and brought to the assessment, the parents/primary caregivers and children/youths answered a questionnaire package on site. The Child Behavior Checklist (CBCL) is a parent-report questionnaire through which the child or adolescent is rated on various behavioral and emotional problems [2]. The Swanson Nolan and Pelham teacher and parent rating scale (SNAP-IV) contains 26 items to measure attention-deficit/hyperactivity disorder (ADHD) and oppositional defiant disorder (ODD) symptoms occurring from childhood to young adulthood [3]. The Inventory of Callous-Unemotional traits (ICU) is a 24-item questionnaire ranging from 0 (not at all true) to 3 (definitely true) and forming three subscales to assess callous and unemotional traits [4]. The Reactive-Proactive Aggression Questionnaire (RPQ) is a 23-item self-report measure of the frequency (never, 0; sometimes, 1; often, 2) of reactive and proactive aggression [5]. IQ was estimated according to four sub-tests derived from the Wechsler Intelligence Scale for Children (WISC-IV) [6] based on block-design similarities, vocabulary, and picture completion. Additionally, the digit span sub-test was assessed. During the study visit, additional tests were conducted that are not relevant to the current investigation. Children/youths were prepared for MR scans, accompanied by their parents/primary caregivers. They were presented with MR sounds and visited a dummy scanner and/or watched an MR information video. If the participants or parent/primary caregivers reported an anxiety rating of >8 on a Visual Analogue Scale (VAS) ranging from 1 (no anxiety) to 10 (very high anxiety) related to entering the MR scanner, the participant was then excluded from further investigation.

If all inclusion criteria were fulfilled, the MR scans took place during a subsequent appointment. The participants answered an MR safety form and a short pre-scanning questionnaire (e.g., regarding tobacco and cannabis consumption, and family genetic disease history). They then practiced the following fMRI tasks on a laptop: a passive-avoidance task, an emotional-matching task, and a stop-signal task. The participants gave a saliva sample right before MRI scanning. They were also asked to use the toilet, and when applicable, pregnancy was ruled out by a urine pregnancy test. The first part of the MRI scanning session included a T1-weighted anatomical image, the three fMRI tasks mentioned above, and an fMRI resting state sequence. After a break for the collection of another saliva sample and a short questionnaire related to the performance in the fMRI tasks, a T1-weighted anatomical scan was followed by two sequences of magnet resonance spectroscopy and diffusion tensor imaging. After scanning, the monetary reward and travel costs were reimbursed. Additionally, participants received an image obtained from their anatomical MR scan. The current study reports the resting state fMRI and psychometric data.

**Data Acquisition**

| **TABLE S1.** Structural MRI scan parameters across sites. | | | | | | | |
| --- | --- | --- | --- | --- | --- | --- | --- |
| **Scanner** | **Site** | **TR/TE/T1 (ms)** | **Flip angle** | **Field of view** | **Matrix RL/AP/slices** | **Voxel size (mm)** | **Acceleration factor** |
| Siemens | Nijmegen | 2300/2.98/900 | 9 | 256 | 212/256/176 | 1.0x1.0x1.2 | 2 |
|  | Mannheim | 2300/2.96/900 | 9 | 256 | 212/256/176 | 1.0x1.0x1.2 | 2 |
|  | Ulm | 2300/2.96/900 | 9 | 256 | 212/256/176 | 1.0x1.0x1.2 | 2 |
|  | Barcelona | 2300/2.98/900 | 9 | 256 | 212/256/176 | 1.0x1.0x1.2 | 2 |
|  | Madrid | 2300/2.98/900 | 9 | 256 | 212/256/176 | 1.0x1.0x1.2 | 2 |
|  | Rome | 2080/2.86/900 | 9 | 256 | 212/256/176 | 1.0x1.0x1.2 | 2 |
| Philips | Groningen | 2450/3.11/900 | 8 | 270 | 256/232/170 | 1.0x1.0x1.0 | 1.8 |
|  | Zurich | 2300/3.11/900 | 9 | 270 | 256/232/170 | 1.0x1.0x1.0 | 1.8 |
| GE | London | 2300/3.02/400 | 11 | 270 | 256/256/196 | 1.0x1.0x1.2 | 1.75 |

| **TABLE S2.** Resting state functional MRI scan parameters across sites. | | | | | | |
| --- | --- | --- | --- | --- | --- | --- |
| **Scanner** | **Site** | **TR/TE1/TE2/TE3 (ms)** | **Number of slices** | **Slice scan order** | **Voxel size (mm)** | **Duration (min)** |
| Siemens | Nijmegen | 2300/12/28.4/44.8 | 33 | descending | 3.8x3.8x3.8 | 8:24 |
|  | Mannheim | 2300/12/29/46 | 33 | descending | 3.8x3.8x3.8 | 8:24 |
|  | Ulm | 2300/31 | 33 | descending | 3.8x3.8x3.8 | 8:23 |
|  | Barcelona | 2300/12 | 33 | descending | 3.8x3.8x3.8 | 8:21 |
|  | Madrid | 2300/13 | 36 | descending | 3.8x3.8x3.8 | 8:24 |
|  | Rome | 2080/30 | 32 | ascending | 3.0x3.0x2.5 | 7:38 |
| Philips | Groningen | 2450/8.01/22.02/36.02 | 45 | descending | 3.5x3.5x3.5 | 10:08 |
|  | Zurich | 2300/13/31/49 | 33 | descending | 3.75x3.75x3.79 | 7:51 |
| GE | London | 2300/11.8/31/48 | 33 | descending interleaved | 3.45x3.45x4.20 | 8:15 |

**Supplementary Results**

Table S3 shows the distribution of demographic characteristics, diagnoses, and clinically relevant aggression scores on behavioral measures across sites for our final sample, including 118 cases and 89 healthy controls. For the seed-based group comparisons and aggression-subtype specific analysis within cases, a statistical threshold of *p* < 0.001 and *p* < 0.008 FWE cluster-level correction (= 0.05 / 6 using additional Bonferroni correction for the number of seeds) for multiple comparisons was applied (see Table S4, Table S6, Table S7). We also conducted sensitivity analyses after exclusion of one site, owing to the small sample size for the case group (*n* < 5) (Table S5). Moreover, after excluding another 10 cases and five controls exceeding the threshold of RMS-FD > 0.5 mm, we conducted sensitivity analyses by rerunning the main seed-based analyses for cases (*n* = 108) compared to controls (*n* = 84) and within cases after correcting for all covariates of no interest (see Table S11 and Table S12). This resulted in comparable case-control differences in resting state functional connectivity and new additional result patterns within cases. In contrast, some aggression-related patterns within cases only survived at a slightly lower uncorrected height threshold of *p* < 0.01 (see Table S11 and Table S12).

The average RMS-FD for cases was 0.12mm (*SD* = 0.17 mm) and for controls 0.09 (*SD* = 0.18mm). In the whole sample, mean RMS-FD was positively correlated with the total score of CU traits (*r* = 0.17, *p* < 0.05), while associations with CBCL rule-breaking, the aggression subscales for callousness, uncaring, and unemotional scores, RA and PA scores, and ADHD inattention hyperactivity and impulsivity scores did not reach significant or suggestive levels. Within cases, there was no significant or trend level correlation between mean RMS-FD and these clinical characteristics reflecting severity.

In our sensitivity analyses, there was no influence of sex on connectivity patterns of cases compared to controls, and in most of the observed subtype-specific patterns within cases (*p* > 0.05), aside from the RA-related right anterior insula seed-based connectivity (*p* < 0.001) and influence of sex at a suggestive level for one instance of uncaring-related left anterior insula seed-based connectivity (*p* = 0.06). Thus, we included this variable as a covariate in all connectivity analyses besides age, IQ, medication, and handedness. For two key cohort variables of sex and age, we performed additional separate analyses.

Thirty-eight cases had an aggression score in the clinical range but no DSM-diagnosis. These cases exhibited comparable reactive aggression scores (*M* = 12.67, *SD* = 5.62) and CU traits (*M* = 33.61, *SD* = 10.01) and lower proactive aggression scores (*M* = 3.91, *SD* = 4.00) compared to cases with a diagnosis (reactive aggression *M* = 12.50, *SD* = 4.87; proactive aggression *M* = 5.27, *SD* = 5.37; CU traits *M* = 33.71, *SD* = 10.25).

As cases and controls differed in average RMS-FD values, we conducted sensitivity analyses controlling for effects of this motion parameter on our case-control differences in connectivity. The PCC connectivity with a left frontal pole cluster yielded comparable statistics (*t*(196) = 5.19, cluster-size *p*-FWE < 0.008, peak *p*-uncorrected < 0.00001, *β* = 0.10), and the left anterior insula connectivity with a left hemispheric cluster extending from the OFC to frontal pole (when additionally controlled for ADHD symptoms) survived as well (*t*(193) = 5.08, cluster-size *p*-FWE < 0.008, peak *p*-uncorrected < 0.00001, *β* = 0.10).

*Results for the whole sample*

Tables 8, 9, and 10 report bivariate correlations of aggression-related scores within cases, the whole sample (cases + controls), and controls, respectively. Additional dimensional analyses were performed for the whole sample and are reported in Table S13 (associations with proactive and reactive aggression) and Table S14 (associations with callous-unemotional traits). Additionally, Figure S1 depicts the results after controlling for all covariates, paralleling the case-related results in the main text. This analysis showed decreased connectivity between the PCC and the angular gyrus for proactive aggression, largely overlapping with increased connectivity of the left amygdala with the precuneus for both proactive and reactive aggression, and hyper-connectivities between the anterior insula (bilaterally) and the pre- and post-central gyri (ICU total score).

*Age as a moderator*

We reran the dimensional analysis with age as a moderator between the connectivity values in the clusters derived from the analysis, controlling for all the covariates without age (Tables S15, S16, and S17) and corresponding scales. A significant interaction between connectivity and age (as a moderator) was found for three effects (only for cases and scores from the Inventory of Callous-Unemotional Traits): the left anterior insula–precuneus and Unemotional traits, the left anterior insula–pre-/post-central gyri and Unemotional traits, and the right anterior insula–post-central gyri and ICU total score (pages 25–26, Figure S2).

*Sex-related effects*

Tables S18−S21 report sex-related connectivity effects (the group by sex interactions along with main effects of sex, the differences between males and females, both in the cases and the whole sample, as well as the sex-specific correlates of PA, RA, and Callous-Unemotional Traits, respectively). The additional tests for the moderating effect of sex on an interaction between the ADHD symptoms (inattention and hyperactivity tested separately) with case/control status (left anterior insula projecting to a cluster in the OFC), and the connectivity values, were not significant (all *p* > 0.05).

| **TABLE S3.** Distribution of demographic characteristics diagnoses and aggression scores across sites. | | | | |
| --- | --- | --- | --- | --- |
|  |  | Cases (*n* = 118) | HC (*n* = 89) |  |
| Nijmegen (*n* = 40) | Age | 13.55 ± 2.59 | 12.64 ± 1.96 |  |
|  | IQ | 100.41 ± 11.83 | 107.86 ± 12.45 |  |
|  | Sex m/f | 14/4 | 14/8 |  |
|  | Medication | 10 | 0 |  |
| Groningen  (*n* = 16) | Age | 14.77 ± 2.54 | 12.39 ± 2.32 |  |
|  | IQ | 101.40 ± 11.63 | 101.85 ± 2.58 |  |
|  | Sex m/f | 8/2 | 2/4 |  |
|  | Medication | 6 | 0 |  |
| Mannheim  (*n* = 33) | Age | 12.78 ± 2.42 | 12.97 ± 3.06 |  |
|  | IQ | 102.16 ± 10.13 | 116.32 ± 7.73 |  |
|  | Sex m/f | 19/3 | 9/2 |  |
|  | Medication | 14 | 0 |  |
| Ulm (*n* = 18) | Age | 10.55 ± 2.38 | 13.59 ± 3.26 |  |
|  | IQ | 103.62 ± 17.55 | 101.41 ± 7.97 |  |
|  | Sex m/f | 5/0 | 4/9 |  |
|  | Medication | 5 | 0 |  |
| London (*n* = 26) | Age | 14.67 ± 2.25 | 13.71 ± 2.05 |  |
|  | IQ | 97.16 ± 10.33 | 111.85 ± 11.01 |  |
|  | Sex m/f | 15/0 | 8/3 |  |
|  | Medication | 12 | 1 |  |
| Barcelona  (*n* = 23) | Age | 12.99 ± 2.82 | 14.94 ± 2.31 |  |
|  | IQ | 102.57 ± 12.10 | 105.70 ± 6.59 |  |
|  | Sex m/f | 10/4 | 5/4 |  |
|  | Medication | 7 | 0 |  |
| Madrid (*n* = 20) | Age | 14.29 ± 2.22 | 14.68 ± 1.84 |  |
|  | IQ | 98.73 ± 9.45 | 105.74 ± 9.31 |  |
|  | Sex m/f | 10/3 | 5/2 |  |
|  | Medication | 10 | 0 |  |
| Zürich  (*n* = 20) | Age | 10.53 ± 1.87 | 11.75 ± 1.49 |  |
|  | IQ | 102.61 ± 11.20 | 99.60 ± 7.87 |  |
|  | Sex m/f | 10/3 | 3/4 |  |
|  | Medication | 2 | 0 |  |
| Rome  (*n* = 11) | Age | 14.37 ± 2.60 | 16.12 ± 1.81 |  |
|  | IQ | 95.84 ± 8.83 | 97.23 ± 2.78 |  |
|  | Sex m/f | 8/8 | 1/2 |  |
|  | Medication | 4 | 0 |  |
| Values are means ± SD or counts. HC = healthy controls.   ODD   CD   ODD + CD   (additional) aggression in a clinical range (T > 70 on aggression subscales in Child Behavior Checklist)  Nijmegen  Groningen Mannheim  Ulm  London  Barcelona Madrid  Zürich Rom | | | | |

| **TABLE S4.**  The clusters and coordinates derived from seed-to-voxel connectivity analyses in cases compared to healthy controls. | | | | | | | | |
| --- | --- | --- | --- | --- | --- | --- | --- | --- |
|  |  | | | | | Peak voxel | | |
|  |  |  |  |  |  | MNI coordinates | | |
|  | Region | Hemisphere |  | Voxels | *z*-value | x | y | z |
| 1 | *PCC Positive Association* - Frontal Pole | L | HC > cases | 225 | 0.10 | -08 | 70 | 12 |
| 2 | *Left Anterior Insula Positive Association* - Posterior Orbital Gyrus | L | HC > cases | 162 | 0.10 | -28 | 32 | -16 |
| Peak voxels are labeled according to the Anatomy toolbox. The statistical threshold for the reported results is *p* < 0.008 FWE cluster-level corrected. *Z*-values represent z-transformed correlation coefficients (Fisher-Z-scores). 1: Group differences corrected for site 2: Group differences corrected for the site and ADHD scores, respectively. Abbreviations: HC, healthy controls; PCC, Posterior Cingulate Cortex. | | | | | | | | |

| **TABLE S5.**  The clusters and coordinates derived from sensitivity analyses of seed-to-voxel connectivity in cases compared to healthy controls after exclusion of one site with a sample size of *n* < 5 for healthy controls. | | | | | | | | |
| --- | --- | --- | --- | --- | --- | --- | --- | --- |
|  |  | | | | | Peak voxel | | |
|  |  |  |  |  |  | MNI coordinates | | |
| Region | | Hemisphere |  | Voxels | *z*-value | x | y | z |
| *PCC Positive Association* - not labeled | | L | HC > cases | 246 | 0.11 | -08 | 68 | 12 |
| Peak voxels are labeled according to the Anatomy toolbox. The statistical threshold for the reported results is *p* < 0.008 FWE cluster-level corrected for multiple comparisons. *Z*-values represent z-transformed correlation coefficients (Fisher-Z-scores). Group differences are corrected for the site. Abbreviations: HC, healthy controls; PCC, Posterior Cingulate Cortex. | | | | | | | | |

| **TABLE S6.** The clusters and coordinates derived from correlation analyses of seed-to-voxel connectivity and proactive and reactive aggression scores within cases. | | | | | | | | |  |
| --- | --- | --- | --- | --- | --- | --- | --- | --- | --- |
|  |  | | | | | Peak voxel | | | |
|  |  |  |  |  |  | MNI coordinates | | | |
|  | Region | Hemisphere |  | Voxels | *z*-value | x | y | z | |
| A | *PCC Positive Association* - Calcarine Gyrus | R | Proactive Aggression | 327 | 0.07 | 04 | -66 | 18 | |
|  | *Left Amygdala  Positive Association* - Precuneus  - Superior Frontal Gyrus | L R |  | 396 237 | 0.04 0.04 | -08 28 | -56 58 | 42 24 | |
|  | *Right Anterior Insula  Positive Association* - Postcentral Gyrus | L |  | 280 | 0.04 | -30 | -42 | 62 | |
|  | *PCC Positive Association* - Fusiform Gyrus | R | Reactive Aggression | 249 | 0.05 | -36 | -28 | -22 | |
|  | *Left Amygdala  Positive Association* - Precuneus | L |  | 864 | 0.05 | -10 | -68 | 56 | |
|  | *Right Anterior Insula  Negative Association* - Calcarine Gyrus | L |  | 393 | -0.05 | -04 | -58 | 10 | |
| B | *Left Amygdala Positive Association* - Midcingulate Cortex | L | Proactive Aggression | 305 | 0.04 | -06 | -42 | 44 | |
|  | *PCC Positive Association* - Fusiform Gyrus | L | Reactive Aggression | 178 | 0.05 | -28 | -20 | -28 | |
|  | *Left Amygdala  Positive Association* - Precuneus | L |  | 900 | 0.05 | -10 | -68 | 56 | |
|  | *Right Anterior Insula Positive Association* - not labeled | R |  | 191 | 0.05 | 24 | 06 | 28 | |
| Peak voxels are labeled according to the Anatomy toolbox. The statistical threshold for the reported results is *p* < 0.008 FWE cluster-level corrected. *Z*-values represent z-transformed correlation coefficients (Fisher-Z-scores). A: Main effects of proactive, respectively reactive aggression corrected for site B: Main effects of proactive, respectively, reactive aggression corrected for the site, age, sex, IQ, medication, and handedness. These effects were not corrected for ADHD symptoms. Abbreviation: PCC, Posterior Cingulate Cortex. | | | | | | | | | |

| **TABLE S7.** The clusters and coordinates derived from correlation analyses of seed-to-voxel connectivity and callous-unemotional traits within cases. | | | | | | | | |  |
| --- | --- | --- | --- | --- | --- | --- | --- | --- | --- |
|  |  | | | | | Peak voxel | | |  |
|  |  |  |  |  |  | MNI coordinates | | |  |
|  | Region | Hemisphere |  | Voxels | *z*-value | x | y | z | |
| A | *Anterior Medial Prefrontal Cortex Positive Association* - Putamen | R | Total score | 241 | 0.05 | 26 | 16 | 00 | |
|  | - Superior Parietal Lobule - Precuneus | L R | Callousness | 785 442 | 0.06 0.06 | -20 12 | -56 -66 | 66 58 | |
|  | *Negative Association -* not labeled | L | Uncaring | 183 | -0.05 | -24 | -10 | 28 | |
|  | *Positive Association* - Cerebellum (VIII) | R | Unemotional | 195 | 0.04 | 30 | -62 | -46 | |
|  | *PCC Positive Association* - Precentral Gyrus | L | Total score | 297 | 0.07 | -54 | 02 | 48 | |
|  | - pMFC - Postcentral Gyrus | L L | Callousness | 379 185 | 0.05 0.05 | -04 -36 | -06 -18 | 60 48 | |
|  | *Left Amygdala Positive Association* - ACC | L | Uncaring | 171 | 0.05 | -14 | 38 | 02 | |
|  | *Left Anterior Insula  Positive Association* - pMFC | L | Total score | 797 | 0.05 | -06 | -16 | 66 | |
|  | - Postcentral Gyrus  - PCC - Precentral Gyrus | L L R | Uncaring | 321 285 238 | 0.06 0.07 0.06 | -20 -02 22 | -32 -42 -30 | 70 30 72 | |
|  | - Precuneus - Angular Gyrus | L L | Unemotional | 544 187 | 0.05 0.05 | -06 -44 | -54 -64 | 16 32 | |
|  | *Right Anterior Insula  Positive Association* - Paracentral Lobule | L | Total score | 1733 | 0.06 | -04 | -20 | 72 | |
|  | - Paracentral Lobule | L | Uncaring | 370 | 0.06 | -06 | -22 | 76 | |
| B | *Anterior Medial Prefrontal Cortex Positive Association* - Inferior Parietal Lobule | L | Total score | 246 | 0.07 | -32 | -56 | 44 | |
|  | - Precuneus - Superior Parietal Lobule | R L | Callousness | 1309 357 | 0.07 0.07 | 12 -28 | -66 -56 | 58 56 | |
|  | - Lobule VIIb (Hem) | R | Uncaring | 188 | 0.06 | 32 | -66 | -60 | |
|  | *PCC Positive Association* - Precentral Gyrus - IFG (pars orbitalis) | L L | Total score | 247 193 | 0.08 0.07 | -54 -46 | 02 12 | 48 00 | |
|  | - Midcingulate Cortex | L | Callousness | 254 | 0.06 | -02 | -14 | 50 | |
|  | *Left Anterior Insula  Positive Association* - pMFC | L | Total score | 858 | 0.06 | -08 | -18 | 64 | |
|  | - Precuneus - Angular Gyrus | L L | Unemotional | 394 171 | 0.06 0.06 | -06 -44 | -54 -64 | 14 32 | |
|  | - Precentral Gyrus - PCC | L L | Uncaring | 552 279 | 0.07 0.08 | 18 -02 | -30 -44 | 72 28 | |
|  | *Right Anterior Insula Positive Association* - not labeled | L | Total score | 422 | 0.06 | -02 | -26 | 70 | |
|  | - Paracentral Lobule | L | Uncaring | 183 | 0.07 | -08 | -22 | 76 | |
| Peak voxels are labeled according to the Anatomy toolbox. The statistical threshold for the reported results is *p* < 0.008 FWE cluster-level corrected. *Z*-values represent z-transformed correlation coefficients (Fisher-Z-scores). A: Main effects of proactive, respectively, reactive aggression corrected for site B: Main effects of proactive, respectively, reactive aggression corrected for site age, sex, IQ, medication, and handedness. These effects were not corrected for ADHD symptoms. Abbreviations: ACC, Anterior Cingulate Cortex; IFG, Inferior Frontal Gyrus; PCC, Posterior Cingulate Cortex; pMFC, posterior Medial Frontal Cortex. | | | | | | | | |  |

| **TABLE S8.**  Bivariate correlations of aggression-related scores within the cases. | | | | | | | | | | |
| --- | --- | --- | --- | --- | --- | --- | --- | --- | --- | --- |
|  | rule-breaking | aggres-sion | ODD | CD | in-attention | hyper-activity | impul-sivity | CU traits | reactive aggres-sion | proactive aggres-sion |
| rule-breaking |  | *r* = 0.47  *p* < .001 | *r* = 0.16   *p >* .05 | *r* = 0.38  *p* < .001 | *r* = 0.12  *p* > .05 | *r* = 0.21  *p* > .05 | *r* = 0.04  *p* > .05 | *r* = 0.16 *p* > .05 | *r* = 0.05 *p* > .05 | *r* = 0.14  *p* > .05 |
| aggres-sion | *r* = 0.47 *p* < .001 |  | *r* = 0.20  *p* < .05 | *r* = 0.21  *p* < .05 | *r* = 0.12  *p* > .05 | *r* = 0.09  *p* > .05 | *r* = 0.14  *p* > .05 | *r* = 0.34  *p* < .001 | *r* = 0.16  *p* > .05 | *r* = 0.22  *p* < .05 |
| ODD | *r* = 0.16  *p >* .05 | *r* = 0.20  *p* < .05 |  | *r* = 0.28  *p* < .01 | *r* = 0.33  *p* < .001 | *r* = 0.28  *p* < .01 | *r* = 0.42  *p* < .001 | *r* = 0.18   *p >* .05 | *r* = 0.07  *p* > .05 | *r* = 0.22  *p* < .05 |
| CD | *r* = 0.47 *p* < .001 | *r* = 0.21  *p* < .05 | *r* = 0.28  *p* < .01 |  | *r* = 0.10  *p* > .05 | *r* = 0.05  *p* > .05 | *r* = 0.07  *p* > .05 | *r* = 0.26  *p* < .01 | *r* = 0.13  *p* > .05 | *r* = 0.29  *p* < .01 |
| in-attention | *r* = 0.12 *p* > .05 | *r* = 0.12  *p* > .05 | *r* = 0.33  *p* < .001 | *r* = 0.10  *p* > .05 |  | *r* = 0.70  *p* < .001 | *r* = 0.68  *p* < .001 | *r* < 0.01  *p* > .05 | *r* = 0.11  *p* > .05 | *r* = -0.12  *p* > .05 |
| hyper-activity | *r* = 0.02  *p >* .05 | *r* = 0.09  *p* > .05 | *r* = 0.28  *p* < .01 | *r* = 0.05  *p* > .05 | *r* = 0.70  *p* < .001 |  | *r* = 0.72  *p* < .001 | *r* = -0.05  *p* > .05 | *r* = 0.06  *p* > .05 | *r* = 0.01  *p* > .05 |
| impul-sivity | *r* = 0.04 *p* > .05 | *r* = 0.14  *p* > .05 | *r* = 0.42  *p* < .001 | *r* = 0.07  *p* > .05 | *r* = 0.68  *p* < .001 | *r* = 0.71  *p* < .001 |  | *r* = -0.08  *p* > .05 | *r* = 0.08  *p* > .05 | *r* < 0.01  *p* > .05 |
| CU traits | *r* = 0.16 *p* > .05 | *r* = 0.34  *p* < .001 | *r* = 0.18   *p >* .05 | *r* = 0.26  *p* < .01 | *r* < 0.01  *p* > .05 | *r* = -0.05  *p* > .05 | *r* = -0.08  *p* > .05 |  | *r* = 0.13  *p* > .05 | *r* = 0.33  *p* = .001 |
| reactive  aggres-sion | *r* = 0.05 *p* > .05 | *r* = 0.16  *p* > .05 | *r* = 0.07  *p* > .05 | *r* = 0.13  *p* > .05 | *r* = 0.11  *p* > .05 | *r* = 0.06  *p* > .05 | *r* = 0.08  *p* > .05 | *r* = 0.13  *p* > .05 |  | *r* = 0.63  *p* < .001 |
| proactive aggres-sion | *r* = 0.14 *p* > .05 | *r* = 0.22  *p* < .05 | *r* = 0.22  *p* < .05 | *r* = 0.29  *p* < .01 | *r* = -0.12  *p* > .05 | *r* = 0.01  *p* > .05 | *r* < 0.01  *p* > .05 | *r* = 0.33  *p* = .001 | *r* = 0.63  *p* < .001 |  |

Rule-breaking and aggression T-scores are derived from the Child Behavior Checklist. ODD CD and ADHD hyperactivity inattention and impulsivity scores according to the Kiddie-Schedule for Affective Disorders and Schizophrenia present and lifetime version. Callous-unemotional (CU) traits total score derived from the parent-reported Inventory of Callous-Unemotional traits reactive and proactive aggression scores according to the self-reported Reactive-Proactive Aggression Questionnaire.

| **TABLE S9.**  Bivariate correlations of aggression-related scores within the whole sample. | | | | | | | | | | |
| --- | --- | --- | --- | --- | --- | --- | --- | --- | --- | --- |
|  | rule-breaking | aggres-sion | ODD | CD | in-attention | hyper-activity | impul-sivity | CU traits | reactive aggres-sion | proactive aggres-sion |
| rule-breaking |  | *r* = 0.75 *p* < .001 | *r* = 0.51  *p <* .001 | *r* = 0.50 *p* < .001 | *r* = 0.43 *p* < .001 | *r* = 0.31 *p* < .001 | *r* = 0.33  *p* < .001 | *r* = 0.32 *p* < .001 | *r* = 0.45 *p* < .001 | *r* = 0.35  *p* < .001 |
| aggres-sion | *r* = 0.75 *p* < .001 |  | *r* = 0.65 *p* < .001 | *r* = 0.43 *p* < .001 | *r* = 0.54  *p* < .001 | *r* = 0.44 *p* < .001 | *r* = 0.47  *p* < .001 | *r* = 0.45 *p* < .001 | *r* = 0.59 *p* < .001 | *r* = 0.46 *p* < .001 |
| ODD | *r* = 0.51  *p <* .001 | *r* = 0.65 *p* < .001 |  | *r* = 0.46 *p* < .001 | *r* = 0.60 *p* < .001 | *r* = 0.52 *p* < .001 | *r* = 0.61 *p* < .001 | *r* = 0.34  *p < .001* | *r* = 0.47 *p* < .001 | *r* = 0.46 *p* < .001 |
| CD | *r* = 0.50 *p* < .001 | *r* = 0.43 *p* < .001 | *r* = 0.46 *p* < .001 |  | *r* = 0.32 *p* < .001 | *r* = 0.24 *p* < .01 | *r* = 0.26 *p* < .001 | *r* = 0.37 *p* < .001 | *r* = 0.33 *p* < .001 | *r* = 0.41 *p* < .001 |
| in-attention | *r* = 0.43 *p* < .001 | *r* = 0.54  *p* < .001 | *r* = 0.60 *p* < .001 | *r* = 0.32 *p* < .001 |  | *r* = 0.78 *p* < .001 | *r* = 0.77 *p* < .001 | *r* = 0.27 *p* < .001 | *r* = 0.42 *p* < .001 | *r* = 0.20 *p* < .01 |
| hyper-activity | *r* = 0.31 *p* < .001 | *r* = 0.44 *p* < .001 | *r* = 0.52 *p* < .001 | *r* = 0.24 *p* < .01 | *r* = 0.78 *p* < .001 |  | *r* = 0.79 *p* < .001 | *r* = 0.18 *p* >.05 | *r* = 0.34 *p* < .001 | *r* = 0.24 *p* < .01 |
| impul-sivity | *r* = 0.33  *p* < .001 | *r* = 0.47  *p* < .001 | *r* = 0.61 *p* < .001 | *r* = 0.26 *p* < .001 | *r* = 0.77 *p* < .001 | *r* = 0.79 *p* < .001 |  | *r* = 0.20 *p* < .01 | *r* = 0.36 *p* < .001 | *r* = 0.24 *p* < .01 |
| CU traits | *r* = 0.32 *p* < .001 | *r* = 0.45 *p* < .001 | *r* = 0.34  *p < .001* | *r* = 0.37 *p* < .001 | *r* = 0.27 *p* < .001 | *r* = 0.18 *p* >.05 | *r* = 0.20 *p* < .01 |  | *r* = 0.47 *p* < .001 | *r* = 0.53 *p* < .001 |
| reactive  aggres-sion | *r* = 0.45 *p* < .001 | *r* = 0.59 *p* < .001 | *r* = 0.47 *p* < .001 | *r* = 0.33 *p* < .001 | *r* = 0.42 *p* < .001 | *r* = 0.34 *p* < .001 | *r* = 0.36 *p* < .001 | *r* = 0.47 *p* < .001 |  | *r* = 0.68 *p* < .001 |
| proactive aggres-sion | *r* = 0.35  *p* < .001 | *r* = 0.46 *p* < .001 | *r* = 0.46 *p* < .001 | *r* = 0.41 *p* < .001 | *r* = 0.20 *p* < .01 | *r* = 0.24 *p* < .01 | *r* = 0.24 *p* < .01 | *r* = 0.53 *p* < .001 | *r* = 0.68 *p* < .001 |  |

Rule-breaking and aggression T-scores are derived from the Child Behavior Checklist. ODD CD and ADHD hyperactivity inattention and impulsivity scores according to the Kiddie-Schedule for Affective Disorders and Schizophrenia present and lifetime version. Callous-unemotional (CU) traits total score derived from the parent-reported Inventory of Callous-Unemotional traits reactive and proactive aggression scores according to the self-reported Reactive-Proactive Aggression Questionnaire.

| **TABLE S10.**  Bivariate correlations of aggression-related scores within controls. | | | | | | | | | | |
| --- | --- | --- | --- | --- | --- | --- | --- | --- | --- | --- |
|  | rule-breaking | aggres-sion | ODD | CD | in-attention | hyper-activity | impul-sivity | CU traits | reactive aggres-sion | proactive aggres-sion |
| rule-breaking |  | *r* = 0.84 *p* < .001 | *X*   . | *r* = 0.27 *p* < .05 | *r* = 0.13 *p* > .05 | *X*   . | *X*   . | *r* = 0.12 *p* > .05 | *r* = 0.31 *p* < .01 | *r* = -0.23 *p* < .05 |
| aggres-sion | *r* = 0.84 *p* < .001 |  | *X*   . | *r* = 0.37 *p* < .001 | *r* = 0.29  *p* > .01 | *X* | *X* | *r* = 0.19  *p* > .05 | *r* = 0.32 *p* < .01 | *r* = -0.09 *p* > .05 |
| ODD | *X* *.* | *X* *.* |  | *X*   . | *X*  . | *X*  . | *X*  . | *X*   *.* | *X*  . | *X*  . |
| CD | *r* = 0.27 *p* < .05 | *r* = 0.37 *p* < .001 | *X* *.* |  | *r* = 0.70  *p* < .001 | *X*  . | *X*  . | *r* = 0.03  *p* > .05 | *r* = 0.24 *p* < .05 | *r* = 0.02 *p* > .05 |
| in-attention | *r* = 0.13 *p* > .05 | *r* = 0.29  *p* > .01 | *X* *.* | *r* = 0.70  *p* < .001 |  | *X*  . | *X*  . | *r* = 0.14 *p* > .05 | *r* = -0.02 *p* > .05 | *r* = -0.06 *p* > .05 |
| hyper-activity | *X* *.* | *X* *.* | *X* *.* | *X*  . | *X*  . |  | *X*  . | *X*  . | *X*  . | *X*   . |
| impul-sivity | *X* *.* | *X* *.* | *X* *.* | *X*  . | *X*  . | *X*  . |  | *X*  . | *X*   . | *X* . |
| CU traits | *r* = 0.12 *p* > .05 | *r* = 0.19  *p* > .05 | *X* *.* | *r* = 0.03  *p* > .05 | *r* = 0.14 *p* > .05 | *X*   . | *X*   . |  | *r* = 0.09 *p* > .05 | *r* = 0.23 *p* < .05 |
| reactive  aggres-sion | *r* = 0.31 *p* < .01 | *r* = 0.32 *p* < .01 | *X* *.* | *r* = 0.24 *p* < .05 | *r* = -0.02 *p* > .05 | *X*   . | *X*   . | *r* = 0.09 *p* > .05 |  | *r* = 0.42 *p* < .001 |
| proactive aggres-sion | *r* = -0.23 *p* < .05 | *r* = -0.09 *p* > .05 | *X* *.* | *r* = 0.02 *p* > .05 | *r* = -0.06 *p* > .05 | *X*   . | *X*   . | *r* = 0.23 *p* < .05 | *r* = 0.42 *p* < .001 |  |

Rule-breaking and aggression T-scores are derived from the Child Behavior Checklist. ODD, CD, and ADHD hyperactivity, inattention, and impulsivity scores according to the Kiddie-Schedule for Affective Disorders and Schizophrenia present and lifetime version. Callous-unemotional (CU) traits total score derived from the parent-reported Inventory of Callous-Unemotional traits reactive and proactive aggression scores according to the self-reported Reactive-Proactive Aggression Questionnaire. X could not be computed.

| **TABLE S11.** Sensitivity Analyses: the clusters and coordinates derived from seed-to-voxel connectivity analyses in cases compared to healthy controls after excluding subjects exceeding the motion threshold of >0.5 mm RMS-FD. | | | | | | | | |
| --- | --- | --- | --- | --- | --- | --- | --- | --- |
|  |  | | | | | Peak voxel | | |
|  |  |  |  |  |  | MNI coordinates | | |
|  | Region | Hemisphere |  | Voxels | *z*-value | x | y | z |
| 1 | *PCC Positive Association* - Middle Orbital Gyrus | L | HC > cases | 251 | 0.10 | -06 | 62 | 02 |
| 2 | *Left Anterior Insula Positive Association* - Inferior Frontal Gyrus pars orbitalis | L | HC > cases | 206 | 0.11 | -30 | 34 | -14 |
| Peak voxels are labeled according to the Anatomy toolbox. The statistical threshold for the reported results is *p* < 0.008 FWE cluster-level corrected. *Z*-values represent z-transformed correlation coefficients (Fisher-Z-scores). 1: Group differences corrected for site 2: Group differences corrected for the site and ADHD scores, respectively. Abbreviations: HC, healthy controls; PCC, Posterior Cingulate Cortex. | | | | | | | | |

| **TABLE S12.**  Sensitivity Analyses: the clusters and coordinates derived from correlation analyses of seed-to-voxel connectivity and reactive aggression scores within cases after excluding cases exceeding the motion threshold of >0.5 mm RMS-FD. | | | | | | | | |  |
| --- | --- | --- | --- | --- | --- | --- | --- | --- | --- |
|  |  | | | | | Peak voxel | | | |
|  |  |  |  |  |  | MNI coordinates | | | |
|  | Region | Hemisphere |  | Voxels | *z*-value | x | y | z | |
|  | *Left Amygdala Positive Association* - Supramarginal Gyrus* - Midcingulate Cortex* - Middle Frontal Gyrus - Supramarginal Gyrus* - Middle Frontal Gyrus* | L L R R R | Proactive Aggression | 895 871 855 632 577 | 0.03 0.03 0.03 0.03 0.03 | -52 -08 34 50 28 | -26 -42 06 -40 62 | 26 44 48 44 22 | |
|  | *PCC Positive Association* - Fusiform Gyrus* | L | Reactive Aggression | 1016 | 0.05 | -28 | -22 | -28 | |
|  | *Left Amygdala  Positive Association* - Precuneus - Angular Gyrus - not labeled - Supramarginal Gyrus | L R R L |  | 1028 300 242 208 | 0.05 0.05 0.05 0.05 | -06 32 34 -56 | -70 -62 04 -28 | 60 54 46 42 | |
|  | *Right Amygdala Positive Association* - Superior Frontal Gyrus | L |  | 156 | 0.05 | -18 | 58 | 24 | |
| Peak voxels are labeled according to the Anatomy Toolbox. The statistical threshold for the reported results is *p* < 0.001 and for * p < 0.01 FWE cluster-level corrected (*p* < 0.008 = 0.05/6 using additional Bonferroni correction for number of seeds) for multiple comparisons. *Z*-values represent z-transformed correlation coefficients (Fisher-Z-scores). Main effects of reactive aggression corrected for the site, age, sex, IQ, medication, and handedness. Abbreviation: PCC, Posterior Cingulate Cortex. | | | | | | | | |  |

| **TABLE S13.** The clusters and coordinates derived from correlation analyses of seed-to-voxel connectivity and proactive and reactive aggression scores within the whole sample (cases + controls). | | | | | | | | | | | | |  |
| --- | --- | --- | --- | --- | --- | --- | --- | --- | --- | --- | --- | --- | --- |
|  |  | | | | | | | | | Peak voxel | | | |
|  |  |  |  |  |  |  |  |  |  |  | | | |
|  |  |  |  |  |  |  |  |  |  | MNI coordinates | | | |
|  | Region | | | Hemisphere | |  | | Voxels | *z*-value | x | y | z | |
| A | | *Left Amygdala*  *Positive Association*  - Precuneus  - Frontal Pole | L/R | | Proactive Aggression | | 1055  260 | | 0.03  0.03 | -08  24 | -56  58 | 42  24 | |
|  | | *PCC*  *Positive Association*  - Frontal Pole  *Left Amygdala*  *Positive Association*  - Precuneus  *Left Anterior Insula*  *Positive Association*  - Angular Gyrus | R  L  R | | Reactive Aggression | | 279  401  272 | | 0.03  0.03  0.04 | 08  -06  68 | 60  -56  -46 | -24  42  28 | |
| B | | *PCC*  *Negative Association*  - Angular Gyrus | R | | Proactive Aggression | | 225 | | -0.07 | 54 | -52 | 30 | |
|  | | *Left Amygdala*  *Positive Association*  - Precuneus | L/R | |  | | 1473 | | 0.04 | -06 | -56 | 42 | |
|  | | *Left Amygdala*  *Positive Association*  - Precuneus | L/R | | Reactive Aggression | | 1179 | | 0.04 | -06 | -70 | 58 | |

Peak voxels are labeled according to the Anatomy Toolbox. The statistical threshold for the reported results is *p* < 0.008 FWE cluster-level corrected. *Z*-values represent z-transformed correlation coefficients (Fisher-Z-scores). A: Main effects of proactive, respectively, reactive aggression corrected for site B: Main effects of proactive, respectively reactive aggression corrected for the site, age, sex, IQ, medication, and handedness. These effects were not corrected for ADHD symptoms. Abbreviation: PCC, Posterior Cingulate Cortex.

| **TABLE S14.** The clusters and coordinates derived from correlation analyses of seed-to-voxel connectivity and callous-unemotional traits within the whole sample (cases + controls). | | | | | | | | | | | | |  |
| --- | --- | --- | --- | --- | --- | --- | --- | --- | --- | --- | --- | --- | --- |
|  |  | | | | | | | | | Peak voxel | | | |
|  |  |  |  |  |  |  |  |  |  | MNI coordinates | | | |
|  | Region | | | Hemisphere | |  | | Voxels | *z*-value | x | y | z | |
|  |  | | |  | |  | |  |  |  |  |  | |
| A | | *Right Anterior Insula*  *Positive Association*  - Precentral/Postcentral gyri | L/R | | Total Score | | 795 | | 0.03 | 14 | -20 | 74 | |
|  | | *Anterior Medial Prefrontal Cortex*  *Positive Association*  - Precuneus | R | | Callousness | | 225 | | 0.04 | 06 | -62 | 56 | |
| B | | *Left Anterior Insula*  *Positive Association*  - Precentral/Postcentral gyri | L/R | | Total Score | | 187 | | 0.04 | -06 | -30 | 60 | |
|  | | *Right Anterior Insula*  *Positive Association*  - Precentral/Postcentral gyri | L/R | |  | | 180 | | 0.04 | -02 | -28 | 68 | |

Peak voxels are labeled according to the Anatomy Toolbox. The statistical threshold for the reported results is *p* < 0.008 FWE cluster-level corrected. *Z*-values represent z-transformed correlation coefficients (Fisher-Z-scores). A: Main effects of proactive, respectively, reactive aggression corrected for site B: Main effects of proactive, respectively reactive aggression corrected for the site, age, sex, IQ, medication, and handedness. These effects were not corrected for ADHD symptoms. Abbreviation: PCC, Posterior Cingulate Cortex.

**FIGURE S1** Distinct connectivity patterns within the whole sample (cases + controls) for Proactive Aggression, Reactive Aggression, and scores from the Inventory of Callous-Unemotional Traits. P, projection cluster; S, seed. Blue indicates negative connectivity, while red indicates positive connectivity. Results are cluster-level FWE-corrected, *p* < 0.008.

| **TABLE S15.** The clusters and coordinates derived from correlation analyses of seed-to-voxel connectivity and proactive and reactive aggression scores within the cases. | | | | | | | | |  |
| --- | --- | --- | --- | --- | --- | --- | --- | --- | --- |
|  |  | | | | | Peak voxel | | | |
|  |  |  |  |  |  | MNI coordinates | | | |
|  | Region | Hemisphere |  | Voxels | *z*-value | x | y | z | |
|  | *PCC Positive Association* - Precuneus | L/R | Proactive Aggression | 173 | 0.07 | 04 | -66 | 18 | |
|  | *Left Amygdala Positive Association* - Midcingulate Cortex | L |  | 307 | 0.04 | -06 | -42 | 44 | |
|  | *PCC Positive Association* - Fusiform Gyrus | L | Reactive Aggression | 273 | 0.05 | -28 | -20 | -28 | |
|  | *Left Amygdala  Positive Association* - Precuneus | L |  | 801 | 0.05 | -10 | -68 | 56 | |
|  | *Right Anterior Insula Positive Association* - not labeled | R |  | 199 | 0.05 | 26 | 10 | 26 | |
| Peak voxels are labeled according to the Anatomy Toolbox. The statistical threshold for the reported results is *p* < 0.008 FWE cluster-level corrected. *Z*-values represent z-transformed correlation coefficients (Fisher-Z-scores). The main effects of proactive, respectively, reactive aggression corrected for the site, sex, IQ, medication, and handedness. This analysis did not control for age, which was further tested as a moderator. These effects were not corrected for ADHD symptoms. Abbreviation: PCC, Posterior Cingulate Cortex. | | | | | | | | | |

| **TABLE S16.** The clusters and coordinates derived from correlation analyses of seed-to-voxel connectivity and callous-unemotional traits within the cases. | | | | | | | | |  |
| --- | --- | --- | --- | --- | --- | --- | --- | --- | --- |
|  |  | | | | | Peak voxel | | |  |
|  |  |  |  |  |  | MNI coordinates | | |  |
|  | Region | Hemisphere |  | Voxels | *z*-value | x | y | z | |
|  | *Anterior Medial Prefrontal Cortex*  *Positive Association* |  | Total score |  |  |  |  |  | |
|  | - Superior Parietal Lobule | L |  | 248 | 0.07 | -32 | -56 | 44 | |
|  | - Frontal Pole | R |  | 172 | 0.08 | 34 | 34 | -08 | |
|  |  |  | Callousness |  |  |  |  |  | |
|  | - Superior Parietal Lobule | R |  | 567 | 0.06 | 42 | -42 | 56 | |
|  | - Superior Parietal Lobule | L |  | 465 | 0.06 | -30 | -62 | 50 | |
|  | - Lateral Occipital Cortex | R |  | 263 | 0.07 | 16 | -60 | 64 | |
|  |  |  | Uncaring |  |  |  |  |  | |
|  | - Cerebellum | R |  | 210 | 0.06 | 32 | -66 | -60 | |
|  | - Supplementary Motor Cortex | R |  | 178 | 0.07 | 04 | 02 | 54 | |
|  | *PCC*  *Positive Association* |  | Callousness |  |  |  |  |  | |
|  | - Midcingulate Cortex | R/L |  | 260 | 0.06 | -02 | -14 | 50 | |
|  |  |  | Uncaring |  |  |  |  |  | |
|  | - Vermis 3 |  |  | 159 | 0.07 | 06 | -42 | -12 | |
|  | - Inferior Temporal Gyrus | L |  | 140 | 0.09 | -48 | -54 | -12 | |
|  | - Insular Cortex | L |  | 137 | 0.06 | -30 | 20 | 0 | |
|  | *Left Anterior Insula*  *Positive Association* |  | Total score |  |  |  |  |  | |
|  | - Pre-/Post-central Gyri | R/L |  | 636 | 0.05 | -06 | -16 | 66 | |
|  |  |  | Uncaring |  |  |  |  |  | |
|  | - Pre-/Post-central Gyri | R/L |  | 394 | 0.07 | 22 | -30 | 72 | |
|  | - Posterior Cingulate Gyrus |  |  | 291 | 0.08 | -02 | -44 | 28 | |
|  |  |  | Unemotional |  |  |  |  |  | |
|  | - Precuneus |  |  | 411 | 0.06 | -06 | -54 | 14 | |
|  | - Lateral Occipital Cortex |  |  | 173 | 0.05 | -44 | -64 | 32 | |
|  | - Pre-/Post-central Gyri |  |  | 135 | 0.06 | -10 | -32 | 68 | |
|  | *Right Anterior Insula*  *Positive Association* |  | Total score |  |  |  |  |  | |
|  | - Pre-/Post-central Gyri | R/L |  | 563 | 0.06 | -02 | -26 | 70 | |
|  |  |  | Uncaring |  |  |  |  |  | |
|  | - Pre-/Post-central Gyri | L |  | 231 | 0.07 | -08 | -22 | 76 | |
|  |  |  |  |  |  |  |  |  | |
| Peak voxels are labeled according to the Anatomy Toolbox. The statistical threshold for the reported results is *p* < 0.008 FWE cluster-level corrected. *Z*-values represent z-transformed correlation coefficients (Fisher-Z-scores). Main effects of callous-unemotional corrected for the site, sex, IQ, medication, and handedness. These effects were not corrected for ADHD symptoms. This analysis did not control for age, which was further tested as a moderator. Abbreviation: PCC, Posterior Cingulate Cortex. | | | | | | | | |  |

| **TABLE S17.** The clusters and coordinates derived from correlation analyses of seed-to-voxel connectivity and proactive/reactive and callous-unemotional dimensions within the whole sample (cases + controls). | | | | | | | | |  |
| --- | --- | --- | --- | --- | --- | --- | --- | --- | --- |
|  |  | | | | | Peak voxel | | |  |
|  |  |  |  |  |  | MNI coordinates | | |  |
|  | Region | Hemisphere |  | Voxels | *z*-value | x | y | z | |
|  | *PCC*  *Negative Association* |  | Proactive Aggression |  |  |  |  |  | |
|  | - Angular Gyrus | R |  | 234 | -0.07 | 54 | -52 | 30 | |
|  | *Left Amygdala*  *Positive Association* |  |  |  |  |  |  |  | |
|  | - Precuneus |  |  | 1542 | 0.04 | -06 | -56 | 42 | |
|  |  |  | Reactive Aggression |  |  |  |  |  | |
|  | - Precuneus |  |  | 1209 | 0.04 | -06 | -70 | 56 | |
|  |  |  |  |  |  |  |  |  | |
|  | *Left Anterior Insula*  *Positive Association* |  | Total score |  |  |  |  |  | |
|  | - Pre-/Post-central Gyri | L/R |  | 180 | 0.04 | -06 | -30 | 60 | |
|  | *Left Anterior Insula*  *Positive Association* |  |  |  |  |  |  |  | |
|  | - Pre-/Post-central Gyri | L/R |  | 181 | 0.04 | -02 | -28 | 68 | |
| Peak voxels are labeled according to the Anatomy Toolbox. The statistical threshold for the reported results is *p* < 0.008 FWE cluster-level corrected. *Z*-values represent z-transformed correlation coefficients (Fisher-Z-scores). The main effects of proactive, respectively, reactive aggression corrected for the site, sex, IQ, medication, and handedness. This analysis did not control for age, which was further tested as a moderator. These effects were not corrected for ADHD symptoms. Abbreviation: PCC, Posterior Cingulate Cortex. | | | | | | | | |  |

**Moderation analysis (age)**

Model : 1

Y : Unemotional trait

X : Connectivity: left anterior insula - precuneus

W : AGE

Sample: Cases

OUTCOME VARIABLE:

Unemotional Trait

Model Summary

R R-sq MSE F df1 df2 p

.4529 .2051 9.6069 7.7419 3.0000 90.0000 .0001

Model

coeff se t p LLCI ULCI

constant 2.4584 1.9614 1.2534 .2133 -1.4383 6.3550

@13_Unem -14.7425 11.2374 -1.3119 .1929 -37.0675 7.5825

AGE .4624 .1524 3.0337 .0032 .1596 .7652

Int_1 1.9540 .8869 2.2032 .0301 .1920 3.7159

Product terms key:

Int_1 : @13_Unem x AGE

Test(s) of highest order unconditional interaction(s):

R2-chng F df1 df2 p

X*W .0429 4.8541 1.0000 90.0000 .0301

----------

Focal predict: X (X)

Mod var: AGE (W)

Conditional effects of the focal predictor at values of the moderator(s):

AGE Effect se t p LLCI ULCI

9.6860 4.1836 3.4370 1.2172 .2267 -2.6445 11.0118

12.9500 10.5614 2.5469 4.1468 .0001 5.5015 15.6212

15.7120 15.9582 3.8821 4.1107 .0001 8.2457 23.6707

**********************************************************************************************

Model : 1

Y : Unemotional trait

X : Connectivity: left anterior insula – pre-/post-central gyri

W : AGE

Sample: cases

OUTCOME VARIABLE:

Unemotional Trait

Model Summary

R R-sq MSE F df1 df2 p

.4577 .2095 9.5539 7.9510 3.0000 90.0000 .0001

Model

coeff se t p LLCI ULCI

constant 3.1406 1.6725 1.8778 .0636 -.1821 6.4633

X -27.0856 16.6731 -1.6245 .1078 -60.2097 6.0385

AGE .3440 .1280 2.6871 .0086 .0897 .5984

Int_1 3.1217 1.3071 2.3882 .0190 .5249 5.7185

Product terms key:

Int_1 : X x AGE

Test(s) of highest order unconditional interaction(s):

R2-chng F df1 df2 p

X*W .0501 5.7037 1.0000 90.0000 .0190

----------

Focal predict: X (X)

Mod var: AGE (W)

Conditional effects of the focal predictor at values of the moderator(s):

AGE Effect se t p LLCI ULCI

9.6860 3.1512 4.8968 .6435 .5215 -6.5773 12.8796

12.9500 13.3404 3.2557 4.0975 .0001 6.8723 19.8085

15.7120 21.9625 5.2645 4.1718 .0001 11.5037 32.4214

**********************************************************************************************

Model : 1

Y : ICU Total Score

X : Connectivity: right anterior insula – pre-/post-central gyri

W : AGE

Sample: Cases

OUTCOME VARIABLE:

ICU Total Score

Model Summary

R R-sq MSE F df1 df2 p

.5823 .3390 72.0930 15.9001 3.0000 93.0000 .0000

Model

coeff se t p LLCI ULCI

constant 26.8069 4.7220 5.6771 .0000 17.4300 36.1838

X -27.8452 43.3567 -.6422 .5223 -113.9431 58.2527

AGE .6406 .3508 1.8261 .0710 -.0560 1.3373

Int_1 6.4402 3.2224 1.9986 .0486 .0411 12.8392

Product terms key:

Int_1 : X x AGE

Test(s) of highest order unconditional interaction(s):

R2-chng F df1 df2 p

X*W .0284 3.9942 1.0000 93.0000 .0486

----------

Focal predict: X (X)

Mod var: AGE (W)

Conditional effects of the focal predictor at values of the moderator(s):

AGE Effect se t p LLCI ULCI

9.7964 35.2452 14.2036 2.4814 .0149 7.0397 63.4508

12.9700 55.6837 9.2489 6.0206 .0000 37.3172 74.0503

15.6688 73.0645 12.3003 5.9401 .0000 48.6385 97.4904

**FIGURE S2** Results of the moderation analysis. Setting age as a moderator of the connectivity effects and dimensional scores showed a significant interaction for unemotional traits and connectivity strength between the left anterior insula and the precuneus, unemotional traits and connectivity strength between the left anterior insula and the pre-/post-central gyri, and ICU total score and connectivity strength between the right anterior insula and the pre-/post-central gyri. All three effects point to symptom scores increasing with age.

| **TABLE S18.** The clusters and coordinates derived from the exploratory seed-to-voxel analysis of the group by sex interactions along with the main effects of sex. | | | | | | | | | |
| --- | --- | --- | --- | --- | --- | --- | --- | --- | --- |
|  |  | | | | | Peak voxel | | | |
|  |  |  |  |  |  | MNI coordinates | | | |
|  | Region | Hemisphere |  | Voxels | *z*-value | x | y | z | |
| 1 | *amPFC Positive Association* - Supramarginal Gyrus | R | interaction group*sex | 241 | 0.18 | 68 | -28 | 26 | |
|  | *amPFC Negative Association* - sLOC | L | main effect of sex | 260 | -0.12 | -36 | -76 | 42 | |
|  | *PCC Positive Association* - PCC (49 voxels)  - not-labeled (128 voxels) | L/R | main effect of sex | 177 | 0.10 | -10 | -40 | 26 | |
|  | *Right Insula Positive Association*  - not labeled | R | main effect of sex | 210 | 0.07 | 08 | 12 | 20 | |
| 2 | *Left insula Negative Association* - STG | R | interaction group*sex | 219 | -0.19 | 62 | 08 | -04 | |
|  | *amPFC Negative Association* - sLOC | L | main effect of sex | 209 | -0.12 | -36 | -76 | 42 | |
|  | *PCC Positive Association* - PCC (64 voxels)  - not-labeled (140 voxels) | L/R | main effect of sex | 204 | 0.11 | -10 | -40 | 22 | |
|  | *Left Insula Negative Association* - Temporal Pole  - SMA | L  L/R | main effect of sex | 167  159 | -0.08  -0.10 | -44 -04 | -10  00 | -28  64 | |
|  | *Right Insula Positive Association* - not-labeled | R | main effect of sex | 174 | 0.08 | 10 | 12 | 20 | |
| The statistical threshold for the results reported is *p* < 0.008 FWE cluster-level corrected. *Z*-values represent z-transformed correlation coefficients. 1: Group differences corrected for site (*n* males = 150; *n* females = 57); 2: Group differences corrected for site, age, IQ, medication and handedness (*n* males = 130; *n* females = 53). Abbreviations: amPFC, anterior medial Prefrontal Cortex; sLOC, superior Lateral Occipital Cortex; PCC, Posterior Cingulate Cortex; SMA, Supplementary Motor Area; STG, Superior Temporal Gyrus. | | | | | | | | |  |

**FIGURE 3.** The group by sex interaction effect. This effect was found for the functional connectivity between the seed in the left insula and a projection cluster in the right superior temporal gyrus (MNI: 62, 08, -04), using a correction for the site, age, IQ, medication, and handedness,

| **TABLE S19.** The clusters and coordinates derived from the exploratory seed-to-voxel analyses in male versus female cases. | | | | | | | | |
| --- | --- | --- | --- | --- | --- | --- | --- | --- |
|  |  | | | | | Peak voxel | | |
|  |  |  |  |  |  | MNI coordinates | | |
|  | Region | Hemisphere |  | Voxels | *z*-value | x | y | z |
| 1 | *PCC Positive Association* - PCC (29 voxels)  - not labeled (177 voxels) | L | males > females | 206 | 0.18 | -10 | -40 | 24 |
| 2 | *PCC Positive Association* - PCC (46 voxels)  - not labeled (175 voxels) | L | males > females | 221 | 0.19 | -12 | -40 | 26 |
| The statistical threshold for the reported results is *p* < 0.008 FWE cluster-level corrected. *Z*-values represent z-transformed correlation coefficients. 1: Group differences corrected for site (*n* males = 99; *n* females = 19); 2: Group differences corrected for site, age, IQ, medication, and handedness (*n* males = 83; *n* females = 17). Abbreviation: PCC, Posterior Cingulate Cortex. | | | | | | | | |

| **TABLE S20.**  The clusters and coordinates derived from the exploratory seed-to-voxel analysis in males versus females in the whole sample (cases + controls). | | | | | | | | |
| --- | --- | --- | --- | --- | --- | --- | --- | --- |
|  |  | | | | | Peak voxel | | |
|  |  |  |  |  |  | MNI coordinates | | |
|  | Region | Hemisphere |  | Voxels | *z*-value | x | y | z |
| 1 | *amPFC Negative Association* - sLOC | L | males > females | 260 | -0.12 | -36 | -76 | 42 |
|  | *PCC Positive Association* - PCC (49 voxels)  - not-labeled (128 voxels) | L/R | males > females | 177 | 0.10 | -10 | -40 | 26 |
|  | *right Insula Positive Association* - Caudate (15 voxels)  - not labeled (195 voxels) | R | males > females | 210 | 0.07 | 08 | 12 | 20 |
| 2 | *amPFC Negative Association* - sLOC | L | males > females | 209 | -0.12 | -36 | -76 | 42 |
|  | *PCC Positive Association* - PCC (64 voxels)  - not-labeled (140 voxels) | L/R | males > females | 204 | 0.11 | 10 | -40 | 22 |
|  | *Left Insula Negative Association* - Temporal Pole  - SMA | L  L | males > females | 167  159 | -0.08  -0.10 | -44  -04 | -10  00 | -28  64 |
|  | *Right Insula Positive Association* - Caudate (18 voxels)  - not labeled (156 voxels) | R | males > females | 174 | 0.08 | 10 | 12 | 20 |
| The statistical threshold for the reported results is *p* < 0.008 FWE cluster-level corrected. *Z*-values represent z-transformed correlation coefficients. 1: Group differences corrected for site (*n* males = 150; *n* females = 57); 2: Group differences corrected for site, age, IQ, medication, and handedness (*n* males = 130; *n* females = 53). Abbreviations: amPFC, anterior medial Prefrontal Cortex; sLOC, superior Lateral Occipital Cortex; PCC, Posterior Cingulate Cortex; SMA, Supplementary Motor Area. | | | | | | | | |

| **TABLE S21.** The clusters and coordinates derived from the exploratory correlation analyses by sex of seed-to-voxel connectivity and traits within the cases. | | | | | | | | |  |
| --- | --- | --- | --- | --- | --- | --- | --- | --- | --- |
|  |  | | | | | Peak voxel | | |  |
|  |  |  |  |  |  | MNI coordinates | | |  |
|  | Region | Hemisphere |  | Voxels | *z*-value | x | y | z | |
|  | *Right Insula*  *Positive Association* |  | Proactive A. |  |  |  |  |  | |
|  | - sLOC | R | m > f | 389 | 0.13 | 40 | -76 | 34 | |
|  | - sLOC | L |  | 166 | 0.14 | -32 | -88 | 36 | |
|  | *Right Insula*  *Negative Association* |  |  |  |  |  |  |  | |
|  | - Precentral Gyrus | R | m > f | 168 | -0.11 | 24 | -16 | 58 | |
|  | *Right Insula*  *Positive Association* |  | Reactive A. |  |  |  |  |  | |
|  | - sLOC | R | m > f | 835 | 0.14 | 38 | -74 | 50 | |
|  | *Right Insula*  *Negative Association* |  |  |  |  |  |  |  | |
|  | - Precentral Gyrus  *Right Insula*  *Positive Association* | R | m > f | 168 | 0.15 | 24 | -16 | 58 | |
|  |  |  |  |  |  |  |  |  |  |
|  |  |  |  |  |  |  |  |  |  |
|  | - Lingual Gyrus | L | m > f | 523 | -0.11 | -10 | -64 | -02 | |
|  | - Intracalcarine Cortex | R |  | 282 | -0.16 | 08 | -68 | -02 | |
|  | - Anterior Cingulate Gyrus | L/R |  | 259 | -0.14 | 02 | -10 | 48 | |
|  | - Parietal Operculum Cortex | L |  | 243 | -0.16 | -50 | -40 | 26 | |
|  | - Posterior Supermarginal Gyrus | R |  | 188 | -0.12 | 62 | -38 | 20 | |
|  | - Thalamus | R |  | 188 | -0.11 | 18 | -16 | 04 | |
|  | - Putamen | R |  | 168 | -0.12 | 28 | -14 | -04 | |
|  | *PCC*  *Positive Association* |  | Callousness |  |  |  |  |  | |
|  | - Middle Temporal Gyrus | L | m > f | 194 | 0.14 | -52 | -60 | 02 | |
|  | *Amygdala Right*  *Negative Association* |  |  |  |  |  |  |  | |
|  | - Occipital Pole | L/R | m > f | 161 | -0.10 | 00 | -84 | 00 | |
|  | *Insula Left*  *Negative Association* |  |  |  |  |  |  |  | |
|  | - Inferior Lateral Occipital Cortex | R | m > f | 657 | -0.11 | 34 | -76 | -08 | |
|  | - Anterior Supramarginal Gyrus | L |  | 250 | -0.14 | -56 | -26 | 30 | |
|  | - Anterior Supramarginal Gyrus | R |  | 177 | -0.17 | 58 | -24 | 22 | |
|  | *Anterior Medial Prefrontal Cortex*  *Negative Association* |  | Uncaring |  |  |  |  |  | |
|  | - Superior Lateral Occipital Cortex | L/R | m > f | 458 | -0.17 | 00 | -72 | 50 | |
|  | *Insula Left*  *Negative Association* |  |  |  |  |  |  |  | |
|  | - Thalamus | R | m > f | 215 | -0.12 | 12 | -06 | 02 | |
|  | *Amygdala Left*  *Negative Association* |  | Unemotional |  |  |  |  |  | |
|  | - Cerebellum | L/R | m > f | 573 | -0.10 | 10 | -80 | -30 | |
| The statistical threshold for the results reported is *p* < 0.008 FWE cluster-level corrected. *Z*-values represent z-transformed correlation coefficients. The effects were corrected for the site, IQ, medication, and handedness. These effects were not corrected for ADHD symptoms. Abbreviations: amPFC, anterior medial Prefrontal Cortex; f, females; m, males; PCC, Posterior Cingulate Cortex; sLOC, superior Lateral Occipital Cortex; SMA, Supplementary Motor Area. | | | | | | | | |  |

**Supplemental References**:

1. Kaufman J, Birmaher B, Brent D, Rao U, Flynn C, Moreci P, et al. Schedule for affective disorders and schizophrenia for school-age children-present and lifetime version (K-SADS-PL): initial reliability and validity data. Journal of the American Academy of Child & Adolescent Psychiatry. 1997;36(7):980-88.

2. Achenbach T, Achenbach T, Achenbach T. Integrative Guide to the 1991 CBCL/4-18, YSR, and TRF Profiles. 1991.

3. Swanson JM. School-based assessments and interventions for ADD students. KC publishing; 1992.

4. Frick PJ. The inventory of callous-unemotional traits. Unpublished rating scale. 2004.

5. Raine A, Dodge K, Loeber R, Gatzke‐Kopp L, Lynam D, Reynolds C, et al. The reactive–proactive aggression questionnaire: Differential correlates of reactive and proactive aggression in adolescent boys. Aggressive Behavior: Official Journal of the International Society for Research on Aggression. 2006;32(2):159-71.

6. Wechsler D. Wechsler Intelligence Scale for Children-(WISC-IV Australian). Sydney: Pearson Clinical and Talent Assessment; 2003.
